# Influence of Environmental Variables on *Gambierdiscus* spp. (Dinophyceae) Growth and Distribution

**DOI:** 10.1371/journal.pone.0153197

**Published:** 2016-04-13

**Authors:** Yixiao Xu, Mindy L. Richlen, Justin D. Liefer, Alison Robertson, David Kulis, Tyler B. Smith, Michael L. Parsons, Donald M. Anderson

**Affiliations:** 1 Key Laboratory of Environment Change and Resources Use in Beibu Gulf, Ministry of Education, Guangxi Teachers Education University, Nanning, Guangxi, China; 2 School of Geographical and Oceanographic Sciences, Nanjing University, Nanjing, Jiangsu, China; 3 Biology Department, Woods Hole Oceanographic Institution, Woods Hole, Massachusetts, United States of America; 4 Department of Geography and Environment, Mount Allison University, Sackville, New Brunswick, Canada; 5 Department of Marine Sciences, University of South Alabama and Dauphin Island Sea Lab, Dauphin Island, Alabama, United States of America; 6 Center for Marine and Environmental Studies, University of the Virgin Islands, St. Thomas, US Virgin Islands, United States of America; 7 Coastal Watershed Institute, Florida Gulf Coast University, Fort Myers, Florida, United States of America; Leibniz Center for Tropical Marine Ecology, GERMANY

## Abstract

Benthic dinoflagellates in the genus *Gambierdiscus* produce the ciguatoxin precursors responsible for the occurrence of ciguatera toxicity. The prevalence of ciguatera toxins in fish has been linked to the presence and distribution of toxin-producing species in coral reef ecosystems, which is largely determined by the presence of suitable benthic habitat and environmental conditions favorable for growth. Here using single factor experiments, we examined the effects of salinity, irradiance, and temperature on growth of 17 strains of *Gambierdiscus* representing eight species/phylotypes (*G*. *belizeanus*, *G*. *caribaeus*, *G*. *carolinianus*, *G*. *carpenteri*, *G*. *pacificus*, *G*. *silvae*, *Gambierdiscus* sp. type 4–5), most of which were established from either Marakei Island, Republic of Kiribati, or St. Thomas, United States Virgin Island (USVI). Comparable to prior studies, growth rates fell within the range of 0–0.48 divisions day^-1^. In the salinity and temperature studies, *Gambierdiscus* responded in a near Gaussian, non-linear manner typical for such studies, with optimal and suboptimal growth occurring in the range of salinities of 25 and 45 and 21.0 and 32.5°C. In the irradiance experiment, no mortality was observed; however, growth rates at 55μmol photons · m^-2^ · s^-1^ were lower than those at 110–400μmol photons · m^-2^ · s^-1^. At the extremes of the environmental conditions tested, growth rates were highly variable, evidenced by large coefficients of variability. However, significant differences in intraspecific growth rates were typically found only at optimal or near-optimal growth conditions. Polynomial regression analyses showed that maximum growth occurred at salinity and temperature levels of 30.1–38.5 and 23.8–29.2°C, respectively. *Gambierdiscus* growth patterns varied among species, and within individual species: *G*. *belizeanus*, *G*. *caribaeus*, *G*. *carpenteri*, and *G*. *pacificus* generally exhibited a wider range of tolerance to environmental conditions, which may explain their broad geographic distribution. In contrast, *G*. *silvae* and *Gambierdiscus* sp. types 4–5 all displayed a comparatively narrow range of tolerance to temperature, salinity, and irradiance.

## Introduction

Ciguatera is a common form of phycotoxin-borne seafood disease caused by the consumption of ciguatoxin-contaminated finfish. It is endemic to the tropical and subtropical Caribbean Sea, and the Pacific, Atlantic, and Indian Oceans. Benthic dinoflagellates in the genus *Gambierdiscus* produce the ciguatoxin precursors responsible for ciguatera toxicity, and their abundance and seasonality has been closely correlated with ciguatera fish poisoning occurrence [1, 2]. Proliferation of *Gambierdiscus* populations is influenced by many environmental factors; amongst them, regimes of temperature, salinity, and irradiance act as determining factors [3–7]. However, the effects of these parameters on *Gambierdiscus* growth, particularly with respect to species-specific responses, are not well known across the multiple species in this genus.

Several early laboratory studies provided initial data in this regard. Both Bomber et al. [3] and Morton et al. [4] conducted growth studies using unialgal cultures to examine *Gambierdiscus* growth responses to temperature, salinity, and irradiance. A major hurdle to interpreting these early results is that *Gambierdiscus* taxonomy was unresolved at that time, and this issue continued until species descriptions were published [8–14]. Previous datasets thus described growth using either *G*. *toxicus* or *Gambierdiscus* sp.; however, many of the strains used could belong to genus of *Fukuyoa* gen. nov. and any of the 11 species in genus of *Gambierdiscus* identified today [8–20] (*G*. *australes*, *G*. *belizeanus*, *G*. *caribaeus*, *G*. *carolinianus*, *G*. *carpenteri*, *G*. *excentricus*, *G*. *pacificus*, *G*. *polynesiensis*, *G*. *scabrosus*/*Gambierdiscus* sp. type 1, *G*. *silvae*/*Gambierdiscus* sp. ribotype 1, *G*. *toxicus*,) and 6 ribotypes (*Gambierdiscus* sp. ribotype2, *Gambierdiscus* sp. type 2–6). These results could even apply to undescribed *Gambierdiscus* species, as morphological and phylogenetic details were not provided. Consequently, it is uncertain whether the growth differences among strains used in these studies resulted from intra- or inter-species variability.

Species-specific *Gambierdiscus* growth data emerged beginning in 2009 [5, 7, 21, 22]. In these studies, inter-specific variability was assessed based on the growth responses of a single strain for each species. Intra-specific variance within each species has yet to be determined, and it is unclear if using multiple strains of *Gambierdiscus* for each species will yield inter-specific growth response patterns similar to those observed previously.

With respect to salinity, *Gambierdiscus* typically attains maximum growth in the salinity range of 25–35, and depending on species/strains, growth is possible over a much wider salinity range (15–41) under laboratory culture conditions [5, 7, 23]. Oceanic waters in areas where *Gambierdiscus* spp. occur are generally restricted to salinities of 34–38; however, *Gambierdiscus* cells have been reported from areas where salinity levels occasionally are outside of this range, such as near river outlets and enclosed water bodies [24, 25].

With regard to irradiance, approximately 10% of full sunlight was previously considered the upper threshold for maximum growth of *Gambierdiscus* [3, 4]. Using irradiance characteristics for multiple species of *Gambierdiscus*, Kibler et al. [5] suggested ~2.5–10% (49–231μmol photons · m^-2^ · s^-1^) of surface irradiance supported maximal growth, with optimal growth extending to 75 m depth in the Caribbean. However, these laboratory results still contrast with field observations of *Gambierdiscus* in habitats exposed to high irradiances, such as sand flats, drifting algae, and detritus [3, 26, 27], where irradiance approaches surface sunlight, yet there are dense cell accumulations. Thus far, only one study indicated that *Gambierdiscus* spp. were notably different in response to irradiance regimes at the species level [5]: *G*. *carolinianus* and *G*. *pacificus* were least adapted to high irradiance and experienced mortality at ~300μmol photons · m^-2^ · s^-1^. To better understand how irradiance affects *Gambierdiscus* growth, data from additional species and strains are needed.

Under experimental culture conditions, *Gambierdiscus* generally achieves maximum growth at 25–31°C, and cannot survive temperatures below ~15–21°C or over ~31–34°C [5, 7, 23, 28, 29]. Field surveys generally agree with this assessment, with *Gambierdiscus* populations and ciguatera incidence primarily reported from environments with a temperature range of 25–30°C [1, 28]. However, recent surveys recorded *Gambierdiscus* cells at extreme temperatures lower and higher than previously reported, e.g, as low as 14°C [30] and ~11°C in the temperate Pacific [31], and in the Red Sea [24], where temperatures can reach or exceed 35°C [32]. Clearly, additional studies on temperature optima and tolerances for *Gambierdiscus* species and strains are needed to help interpret these reports, as well as to enhance our understanding of the distribution and seasonality of species within this genus in the context of climate change.

This study sought to determine the optimal conditions for growth and the tolerances to temperature (16–38°C), salinity (10–60) and irradiance (55–400μmol photons · m^-2^ · s^-1^) of eight *Gambierdiscus* species/phylotypes: *G*. *belizeanus*, *G*. *caribaeus*, *G*. *carolinianus*, *G*. *carpenteri*, *G*. *pacificus*, *G*. *silvae*, *Gambierdiscus* sp. type 4 and *Gambierdiscus* sp. type 5. Multiple strains were examined for five of the eight species tested. Furthermore, this study represents the first examination of the growth responses of *G*. *silvae*, and two Pacific ribotypes (*Gambierdiscus* sp. type 4 and type 5). The growth responses determined from laboratory experiments were compared with the distribution of *Gambierdiscus* and ciguatera occurrence in an effort to better understand how these environmental variables influence *Gambierdiscus* growth and distribution.

## Materials and Methods

### Ethics statement

The locations of the field studies are not privately owned or protected. No activity during field study involved any endangered species or protected species. Thus no specific permissions were required for all locations/activities for this study.

### Source of *Gambierdiscus* isolates

In the Caribbean, individual *Gambierdiscus* cells were obtained from the macroalga *Dictyota* spp. at St. Thomas, USVI (18° 20' 7.30'' N, 64° 57' 12.24'' W), with the exception of *G*. *carpenteri*, which was collected from *Halimeda monile* at Long Key, Florida Keys (24° 49' 36.70'' N, 80° 48' 51.53'' W). In the Pacific, cells were obtained from *Halimeda* spp. and coral rubble at Marakei Island, Republic of Kiribati (2° 0' 0'' N, 173° 16' 0'' E). Macroalgae sample processing, *Gambierdiscus* isolation, and culture establishment procedures were similar to Xu et al. [19]. Isolates were maintained in modified K medium in which Tris buffer and silicate were omitted, with incubation at 23°C, salinity of 32, 100μmol photons · m^-2^ · s^-1^ of light, and 12:12h light: dark photoperiod. Cultures were maintained for 3–12 months to acclimate to laboratory conditions before they were used in the growth experiments. This study included a total of six *Gambierdiscus* species and two *Gambierdiscus* ribotypes, the latter of which may represent undescribed species. Details regarding the isolates are listed in Table 1.

**Table 1:** Species identification and geographic origin of the seventeen *Gambierdiscus* strains used in growth rate experiments.

Species	Strain	Collection date	Origin
*G*. *belizeanus*	BP Mar10_6	Mar, 2010	St. Thomas, USVI
*G*. *belizeanus*	BP Mar10_7	Mar, 2010	St. Thomas, USVI
*G*. *belizeanus*	BP Mar10_22	Mar, 2010	St. Thomas, USVI
*G*. *belizeanus*	FC Dec10_13	Dec, 2010	St. Thomas, USVI
*G*. *caribaeus*	BP Aug08	Aug, 2008	St. Thomas, USVI
*G*. *caribaeus*	FC Nov09_4	Nov, 2009	St. Thomas, USVI
*G*. *caribaeus*	SH Nov09_3	Nov, 2009	St. Thomas, USVI
*G*. *carolinianus*	SH Mar10_12	Mar, 2010	St. Thomas, USVI
*G*. *carolinianus*	BB Apr10_3	Apr, 2010	St. Thomas, USVI
*G*. *carolinianus*	BP May10_1	May, 2010	St. Thomas, USVI
*G*. *carpenteri*	KML1	Mar, 2011	Long Key, Florida Keys
*G*. *pacificus*	3S0509-27	May, 2011	Marakei Island, Kiribati
*G*. *pacificus*	3S0510-19	May, 2011	Marakei Island, Kiribati
*G*. *silvae*	FC May10_9	May, 2010	St. Thomas, USVI
*Gambierdiscus* sp. type 4	1D0509-16	May, 2011	Marakei Island, Kiribati
*Gambierdiscus* sp. type 4	1D0510-22	May, 2011	Marakei Island, Kiribati
*Gambierdiscus* sp. type 5	DS0511-03	May, 2011	Marakei Island, Kiribati

USVI: United States Virgin Islands

### *In vivo* fluorescence and growth rate

*Gambierdiscus* growth was assessed by *in vivo* fluorescence using a 10-AU Fluorometer (Turner Designs, USA). Previous studies confirmed a linear correlation between *in vivo* fluorescence (relative fluorescence units or RFU) and cell densities (biomass), in which increasing fluorescence was associated with increasing cell numbers rather than an increase in fluorescence per cell [3–5, 7]. Fluorescence measurements were used to plot fluorescence (log) vs. time; the exponential growth phase portion of this curve was then utilized to calculate growth rates following the equation defined in Guillard [33],

$$\mu \;\left({\text{division day}}^{-1}\right)=\frac{\text{ln }(N_{1}\;/\;N_{0})}{0.6931\;\left(t_{1}-t_{0}\right)}$$

in which *μ* (division · day^-1^) is the growth rate, and *N*_1_ and *N*_0_ represent the fluorescence at times *t*_1_ and *t*_0_, respectively. In all experiments, a total of four sequential transfers were performed: the first transfer was conducted to allow the culture to acclimate to the environmental conditions, and data from the second-fourth transfers were collected to determine the growth rate.

Culture fluorescence was measured twice every week at 3- to 4-days intervals. To reduce error during the collection of fluorescence measurements, cultures were mixed fully prior to fluorescence reading. This process differed among species; for *G*. *carolinianus* and *Gambierdiscus* sp. type 4, vortexing was required to resuspend the clumped cells; for the remaining cultures, hand-mixing was sufficient to resuspend cells evenly. All species except *G*. *belizeanus* were transferred when fluorescence was >50 RFU. In the case of *G*. *belizeanus* preliminary results indicated that cells grew poorly when transferred at <70 RFU, and these cultures were thus transferred when cells were at fluorescence >80 RFU.

### Salinity experiments

Salinity experiments were carried out in a model I-35 LLVL Percival incubator (Perry, Iowa, USA) under a constant average temperature of 27°C, and the aforementioned irradiance and photoperiod intervals. Salinity levels ranged from 10–60 (10, 15, 20, 25, 30, 35, 40, 45, 50, 55, and 60), which were created by adding Milli-Q water into natural filtered seawater (salinity 32) to reduce salinity, or adding sea salt evaporated from 0.2 μm filtered natural seawater to increase salinity. This salinity-treated medium was then autoclaved for 45 minutes in Teflon bottles. After autoclaving, the salinity was measured again with a hand-held refractometer and enriched to produce modified K medium where the tris component was omitted. The procedures for determining growth were as described for the temperature experiments. Culture tubes in salinity experiments were placed randomly in the incubator to mitigate micro-environmental differences.

### Irradiance experiments

Irradiance experiments were performed in an incubator with a constant mean temperature of 27°C. Irradiance levels of 55, 110, 200, and 400μmol photons·m^-2^·s^-1^ were established using four equidistant shelves in the incubator. Between 0–6 cool white full spectrum fluorescent bulbs were installed in each shelf, and covered by 0–2 layers of nylon window screens to achieve the desired irradiance levels. Irradiance received by the culture tubes was measured using Digital Scalar Irradiance Meter (Model #: QSP-170, Biospherical Instruments Inc., CA, USA) with probe QSL-100 (Serial #: 1275, Biospherical Instruments Inc.). Culture tubes were placed in random locations in the incubator to once again minimize micro environmental disparity.

### Temperature experiments

Temperature experiments were conducted in a temperature gradient bar [34] constructed from an aluminum plate (122cm x 23cm x 5cm) with 120 25mm diameter holes evenly spaced in 20 columns, each with six positions for replicates. Heating was provided by a 300 watt cartridge probe inserted in one end of the bar and cooling water was supplied by a circulating water bath (model # 1156D, VWR, PA, USA) through the opposite end to set up a thermal gradient ranging from 16–38°C, which included a total of 17 successive temperatures: 16.0, 17.5, 18.5, 20.0, 21.0, 22.5, 24.0, 25.5, 27.0, 28.5, 30.0, 31.0, 32.5, 34.0, 35.0, 36.0 and 38.0°C. Lighting from cool white fluorescent bulbs was supplied below the bar, which provided an average irradiance level of 200μmol photons · m^-2^ · s^-1^, under a photoperiod cycle of 12h:12h light: dark. The first transfer was used to acclimate cultures to temperature conditions, and growth rates were calculated from three additional sequential transfers over the culture’s exponential phase of growth.

### Data analysis

To assess growth variability and statistical differences in growth rates within each *Gambierdiscus* species in all experiments, coefficient of variation (CV) among conspecific strains was calculated; depending on normality and homoscedasticity of growth rate data, a one-way analysis of variance (ANOVA) (normality, homoscedasticity), Welch’s ANOVA (normality, heteroscedasticity), Mann-Whitney Test (2 Independent Samples, non-normality and homoscedasticity) and Kruskal-Wallis H Test (K≥3 Independent Samples, non-normality and homoscedasticity) were performed using SPSS statistics 21 (SPSS Inc., IL, USA). In addition, to better understand the growth potential of each *Gambierdiscus* species/strain, growth rate and salinity, and growth rate and temperature were polynomial fitted using OriginPro 9.0.0 (OriginLab Corporation, MA, USA). The polynomial equations were further analyzed using Matlab R2013a 8.1.0.604 (Mathworks Inc., MA, USA) to calculate the characteristic points where growth maxima, optima, and limitation occurred.

The growth characteristics were described by the following parameters similarly defined in Kibler et al. [5]: *μ*_*m*_, maximum growth rate; *T*_m_/*S*_m_, temperature/salinity of maximum growth; *T*_opt_/*S*_opt_, temperature/salinity of optimum growth range (*μ* ≥ 0.8 × *μ*_m_); *T*_o_/*S*_o_, the lower temperature/salinity limit for growth, and *T*_u_/*S*_u_, the upper temperature/salinity limit for growth. Due to data limitations, polynomial regressive analysis was not performed in the irradiance experiment.

## Results

### Salinity

#### Growth rate

**Fig 1:**
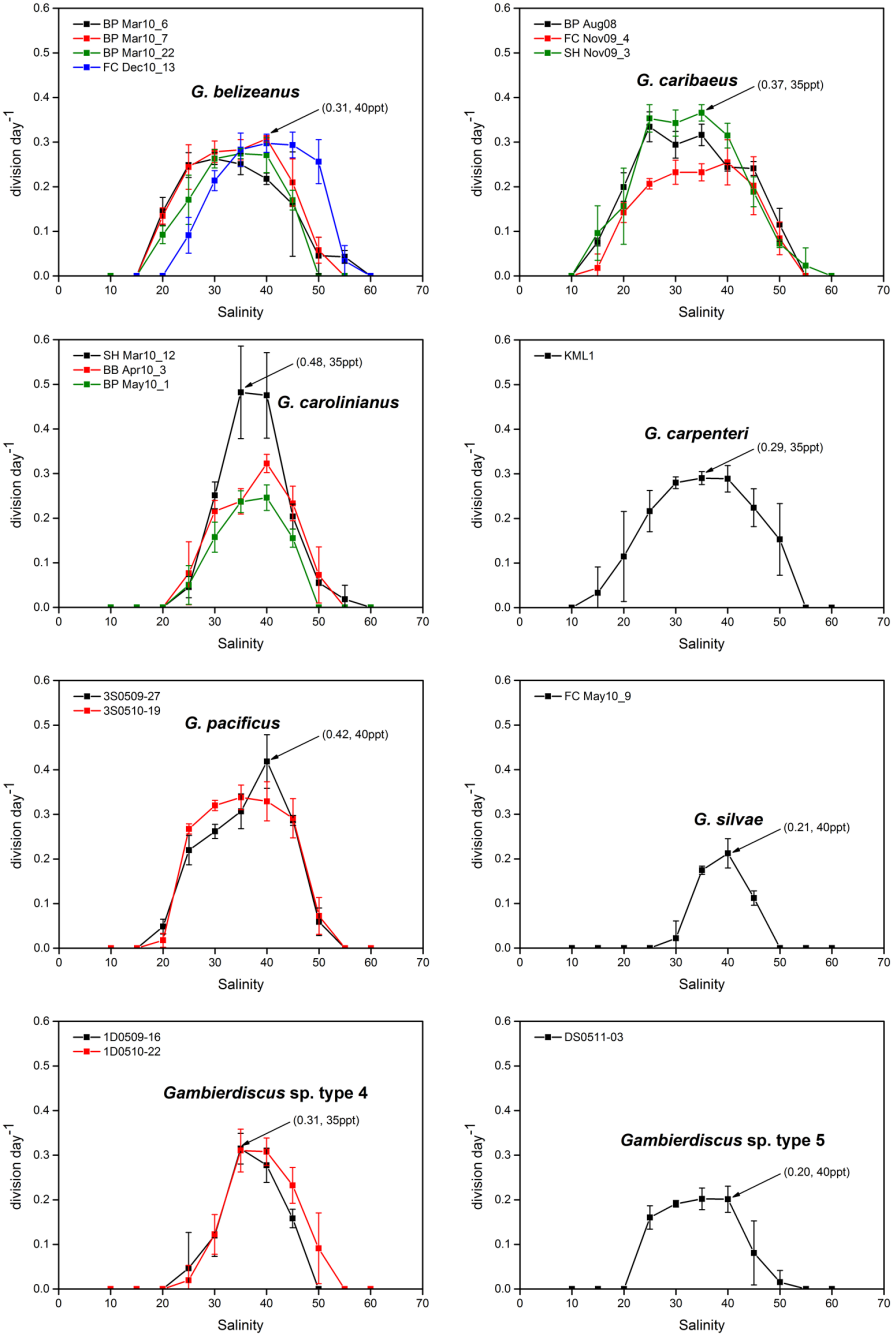
Growth rates of *Gambierdiscus* strains versus culture salinity of 10–60. Each point is the mean of triplicate measurements, and the bars represent standard deviation (SD). Black arrows represent the maximum growth rate for a species.

**Fig 2:**
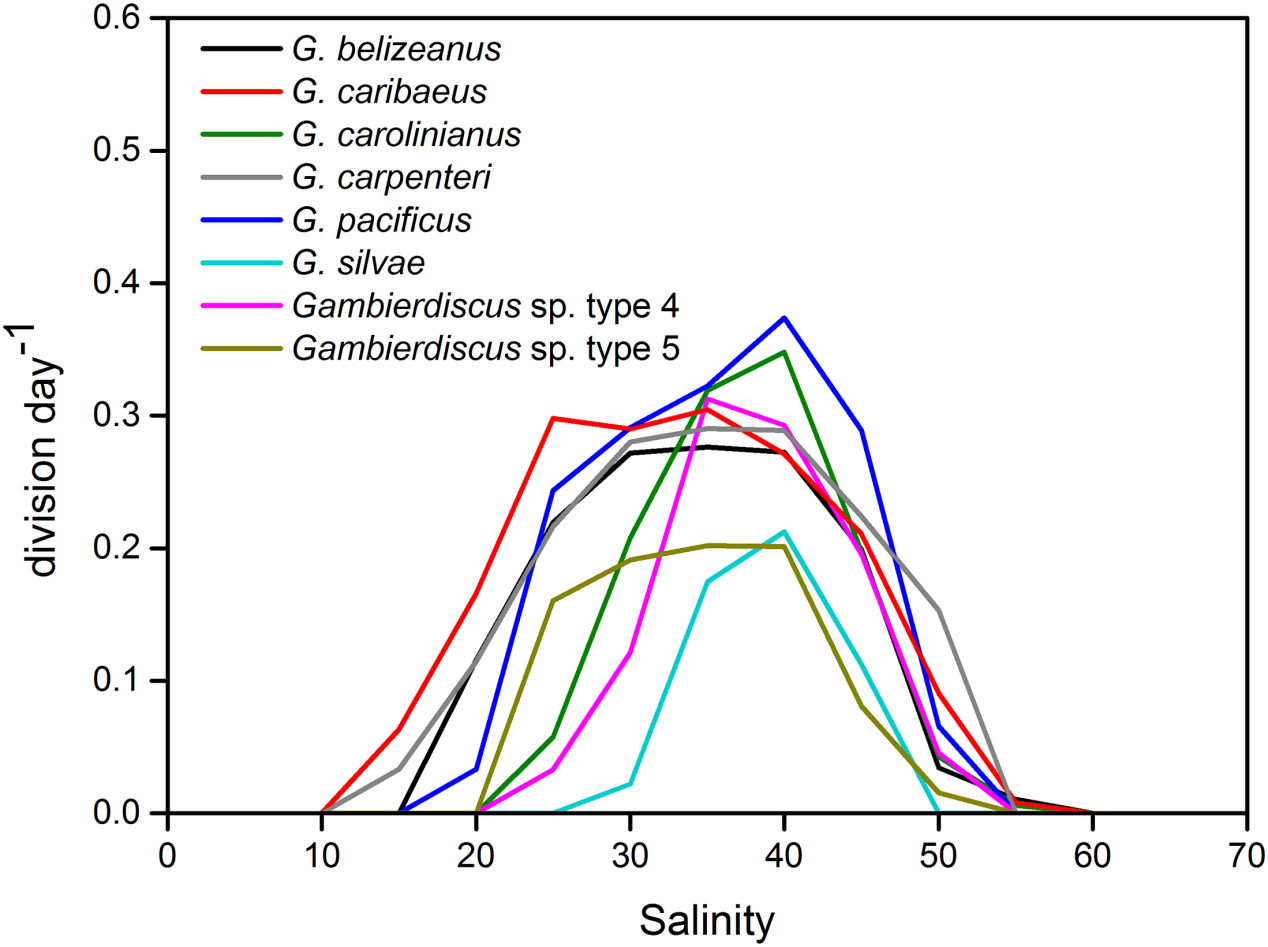
Average growth response of *Gambierdiscus* species to salinity of 10–60. For ease of viewing, error bars shown in Fig 1 are omitted here.

Growth responses of *Gambierdiscus* spp. to different salinity at a constant mean temperature of 27°C are shown in Figs 1 and 2. *Gambierdiscus* growth curves showed a general increase in growth rate with increasing salinity values, followed by a decline at high salinities (Fig 1). *Gambierdiscus* cells generally did not grow in salinities of ≤20 or ≥50; however, certain strains of *G*. *caribaeus* and *G*. *carpenteri* survived at a salinities of ~15 and >50, and strains of *G*. *belizeanus* (BP Mar10_6), *G*. *caribaeus* (SH Nov09_3), and *G*. *carolinianus* (SH Mar10_12) exhibited low growth at these salinity levels (Fig 1).

Growth rates during the salinity studies ranged from no growth to 0.48 division day^-1^; maximum growth rates for each clone were observed at salinities of 30–40, but differed among species as shown in Fig 1. *Gambierdiscus carolinianus* and *G*. *pacificus* exhibited the highest average maximum growth rates, whereas *Gambierdiscus* sp. type 5 had the lowest. *Gambierdiscus caribaeus* and *G*. *carpenteri* exhibited broad tolerance to salinity compared with the other species examined in this study (Fig 2).

#### Intraspecific variation

The CV values calculated for *G*. *belizeanus*, *G*. *caribaeus*, *G*. *carolinianus*, *G*. *pacificus*, and *Gambierdiscus* sp. type 4 ranged from 0.9–200.0% (salinity of 20–55, 15–55, 25–55, 20–50, and 25–50) (Table 2). High CV values were found at the upper and lower extreme salinity levels, and statistically significant differences in growth mostly occurred at the mid range of salinities of 25–45 (Table 2).

**Table 2:** *Gambierdiscus* strain numbers, growth rate, coefficient of variation among strains, and intraspecific differences for each species and salinity (10–60).

Species	Salinity	Number of strains	Growth rate (mean ± SD day^-1^)	Coefficient of variation (%)	p value
*G*. *belizeanus*	10	4	no growth		
*G*. *belizeanus*	15	4	no growth		
*G*. *belizeanus*	20	4	0.12 ± 0.03	24.2	0.106
*G*. *belizeanus*	25	4	0.22 ± 0.04	16.3	0.158
*G*. *belizeanus*	30	4	0.27 ± 0.01	3.8	0.686
*G*. *belizeanus*	35	4	0.28 ± 0.02	7.1	0.106
*G*. *belizeanus*	40	4	0.27 ± 0.04	14.5	**0.012**
*G*. *belizeanus*	45	4	0.20 ± 0.04	21.8	0.380
*G*. *belizeanus*	50	4	0.03 ± 0.02	72.7	0.194
*G*. *belizeanus*	55	4	0.01 ± 0.02	200.0	**0.013**
*G*. *belizeanus*	60	4	no growth		
*G*. *caribaeus*	10	3	no growth		
*G*. *caribaeus*	15	3	0.06 ± 0.04	64.0	0.144
*G*. *caribaeus*	20	3	0.17 ± 0.03	17.9	0.193
*G*. *caribaeus*	25	3	0.30 ± 0.08	26.7	**0.001**
*G*. *caribaeus*	30	3	0.29 ± 0.06	19.1	**0.010**
*G*. *caribaeus*	35	3	0.30 ± 0.07	22.1	**0.001**
*G*. *caribaeus*	40	3	0.27 ± 0.04	14.0	0.085
*G*. *caribaeus*	45	3	0.21 ± 0.03	12.9	0.368
*G*. *caribaeus*	50	3	0.09 ± 0.02	24.2	0.285
*G*. *caribaeus*	55	3	0.01 ± 0.01	173.2	0.368
*G*. *caribaeus*	60	3	no growth		
*G*. *carolinianus*	10	3	no growth		
*G*. *carolinianus*	15	3	no growth		
*G*. *carolinianus*	20	3	no growth		
*G*. *carolinianus*	25	3	0.06 ± 0.02	29.1	0.725
*G*. *carolinianus*	30	3	0.21 ± 0.05	22.7	**0.022**
*G*. *carolinianus*	35	3	0.32 ± 0.14	44.3	**0.005**
*G*. *carolinianus*	40	3	0.35 ± 0.12	33.6	**0.008**
*G*. *carolinianus*	45	3	0.20 ± 0.04	19.9	**0.049**
*G*. *carolinianus*	50	3	0.04 ± 0.04	88.9	0.126
*G*. *carolinianus*	55	3	0.01 ± 0.01	173.2	0.368
*G*. *carolinianus*	60	3	no growth		
*G*. *pacificus*	10	2	no growth		
*G*. *pacificus*	15	2	no growth		
*G*. *pacificus*	20	2	0.03 ± 0.02	65.6	0.079
*G*. *pacificus*	25	2	0.24 ± 0.03	13.8	0.078
*G*. *pacificus*	30	2	0.29 ± 0.04	14.1	**0.007**
*G*. *pacificus*	35	2	0.32 ± 0.02	6.9	0.320
*G*. *pacificus*	40	2	0.37 ± 0.06	16.9	0.106
*G*. *pacificus*	45	2	0.29 ± 0.00	1.2	0.864
*G*. *pacificus*	50	2	0.07 ± 0.01	14.2	0.680
*G*. *pacificus*	55	2	no growth		
*G*. *pacificus*	60	2	no growth		
*Gambierdiscus* sp. type 4	10	2	no growth		
*Gambierdiscus* sp. type 4	15	2	no growth		
*Gambierdiscus* sp. type 4	20	2	no growth		
*Gambierdiscus* sp. type 4	25	2	0.03 ± 0.02	57.8	0.621
*Gambierdiscus* sp. type 4	30	2	0.12 ± 0.00	1.5	0.949
*Gambierdiscus* sp. type 4	35	2	0.31 ± 0.00	0.9	0.910
*Gambierdiscus* sp. type 4	40	2	0.29 ± 0.02	7.3	0.345
*Gambierdiscus* sp. type 4	45	2	0.20 ± 0.05	26.9	**0.047**
*Gambierdiscus* sp. type 4	50	2	0.05 ± 0.06	141.4	0.121
*Gambierdiscus* sp. type 4	55	2	no growth		
*Gambierdiscus* sp. type 4	60	2	no growth		

Salinities at which intraspecific variation was significant are listed in bold (α < 0.05).

#### Polynomial regression analysis

**Fig 3:**
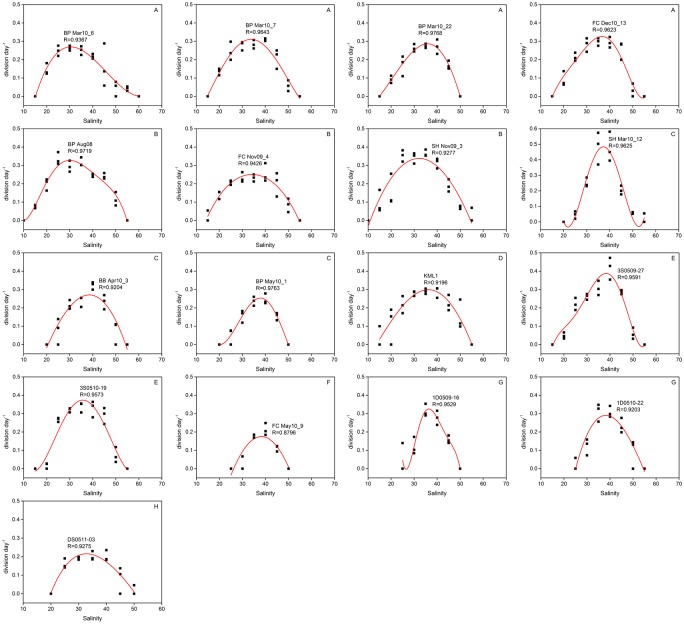
*Gambierdiscus* growth rate responses (black squares) to salinities of 10–60 and simulated growth curves (red line) by polynomial regressive analysis. (A) *G*. *belizeanus* (BP Mar10_6, BP Mar10_7, BP Mar10_22, FC Dec10_13), (B) *G*. *caribaeus* (BP Aug08, FC Nov09_4, SH Nov09_3), (C) *G*. *carolinianus* (SH Mar10_12, BB Apr10_3, BP May10_1), (D) *G*. *carpenteri* (KML1), (E) *G*. *pacificus* (3S0509-27, 3S0510-19), (F) *G*. *silvae* (FC May10_9), (G) *Gambierdiscus* sp. type 4 (1D0509-16, 1D0510-22), and (H) *Gambierdiscus* sp. type 5 (DS0511-03).

To assess *Gambierdiscus* growth potential at each salinity, growth rate and salinity were described using the polynomial equation: Y = A + B_1_X + B_2_X^2^ + … + B_n_X^n^. Here, X and Y represent salinity and growth rate, respectively. Most strains were fitted to 3^rd^ to 5^th^ order polynomial curves with an R value >0.9 (Fig 3, S1 Table).

Polynomial curves for salinity experiments exhibited a relatively symmetrical bell shape (Fig 3). In the salinity study, *μ*_m_ varied from 0.18–0.47 divisions day^-1^; *S*_m_ fell in the salinity range of 30.1–38.5 (Table 3). The major difference in salinity response was observed in the optimum salinity range (*S*_opt_), and the ability to maintain growth or survive at extreme salinities (*S*_o_ and *S*_u_). Generally speaking, strains of *G*. *caribaeus* and *G*. *carpenteri* had a broad *S*_opt_ and tolerated extreme salinities, whereas strains of *G*. *belizeanus* were tolerant to hypersaline conditions. In contrast, strains of *G*. *carolinianus*, *G*. *silvae*, and *Gambierdiscus* sp. types 4–5 were sensitive to extreme salinities.

**Table 3:** *Gambierdiscus* species growth parameters at salinities of 10–60. Individual growth rate measurements were fitted to polynomial curves. The polynomial equations were used for growth parameter estimation: *μ*_*m*_, maximum growth rate; *S*_m_, salinity of maximum growth; *S*_opt_, salinity of optimum growth range (*μ*≥0.8×*μ*_m_); *S*_o_, the lower salinity limit for growth; *S*_u_, the upper salinity limit for growth.

Strain	Species	*μ*_*max*_	*S*_m_	*S*_opt_	*S*_opt_ range	*S*_o_	*S*_u_
BP Mar10_6	*G*. *belizeanus*	0.27	30.3	23.0–38.9	15.9	15.1	57.0
BP Mar10_7	*G*. *belizeanus*	0.31	33.9	26.1–41.9	15.8	14.8	54.6
BP Mar10_22	*G*. *belizeanus*	0.29	35.9	28.3–42.4	14.1	15.0	50.0
FC Dec10_13	*G*. *belizeanus*	0.33	36.6	29.3–42.8	13.5	15.2	52.8
BP Aug08	*G*. *caribaeus*	0.33	30.1	22.6–40.1	17.5	10.4	55.1
FC Nov09_4	*G*. *caribaeus*	0.25	34.4	24.8–44.0	19.2	14.2	54.9
SH Nov09_3	*G*. *caribaeus*	0.34	32.2	22.5–42.0	19.5	10.9	54.5
SH Mar10_12	*G*. *carolinianus*	0.47	37.2	32.4–42.0	9.6	24	50.2
BB Apr10_3	*G*. *carolinianus*	0.27	38.5	30.8–45.8	15.0	20.6	54.3
BP May10_1	*G*. *carolinianus*	0.25	37.8	32.4–42.9	10.5	20.7	49.9
KML1	*G*. *carpenteri*	0.29	36.0	27.0–44.5	17.5	13.6	54.8
3S0509-27	*G*. *pacificus*	0.30	36.9	31.0–41.7	10.7	15.5	47.6
3S0510-19	*G*. *pacificus*	0.37	35.6	28.8–42.3	13.5	16.5	53.8
FC May10_9	*G*. *silvae*	0.18	38.3	32.8–43.7	10.9	26.1	50.4
1D0509-16	*Gambierdiscus* sp. type 4	0.32	36.4	32.9–40.6	7.7	27.2	49.7
1D0510-22	*Gambierdiscus* sp. type 4	0.28	38.1	32.0–44.7	12.7	25.1	54.1
DS0511-03	*Gambierdiscus* sp. type 5	0.22	32.9	26.6–40.1	13.5	20.0	50.1

### Irradiance

#### Growth rate

**Fig 4:**
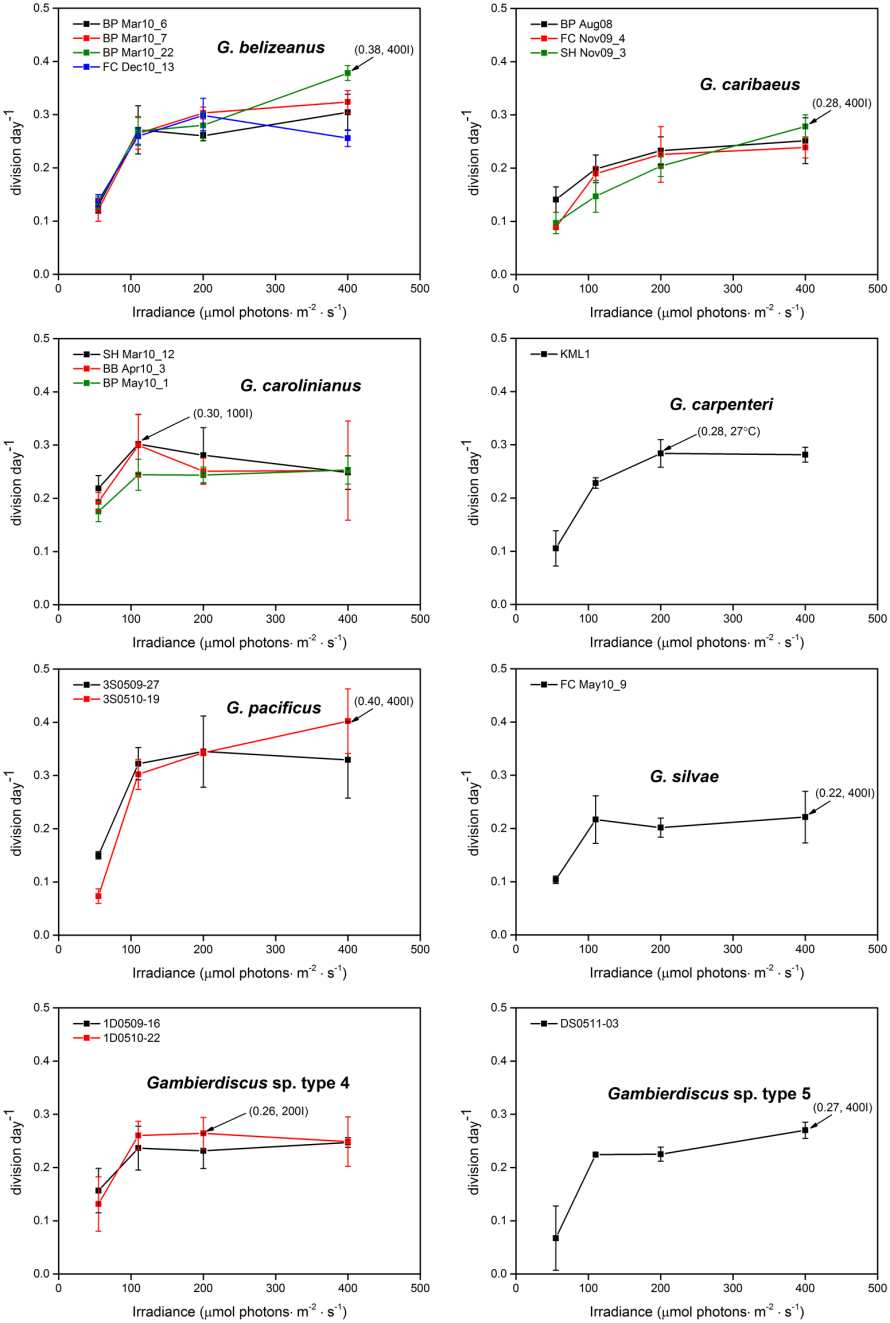
Growth rates of *Gambierdiscus* strains versus culture irradiance of 55–400μmol photons · m^-2^ · s^-1^. Each point is the mean of triplicate measurements, and the bars represent standard deviation (SD). Black arrows represent the maximum growth rate for a species.

Net growth was observed for all *Gambierdiscus* strains across the range of irradiances tested (55–400μmol photons · m^-2^ · s^-1^) under a constant mean temperature of 27°C and a salinity of 32. Growth responses to varying irradiance were similar for all 17 strains; i.e., growth was slow at 55μmol photons · m^-2^ · s^-1^ and generally plateaued in the range of 110–400μmol photons · m^-2^ · s^-1^. No obvious decline was observed at 400μmol photons · m^-2^ · s^-1^ (Fig 4).

**Fig 5:**
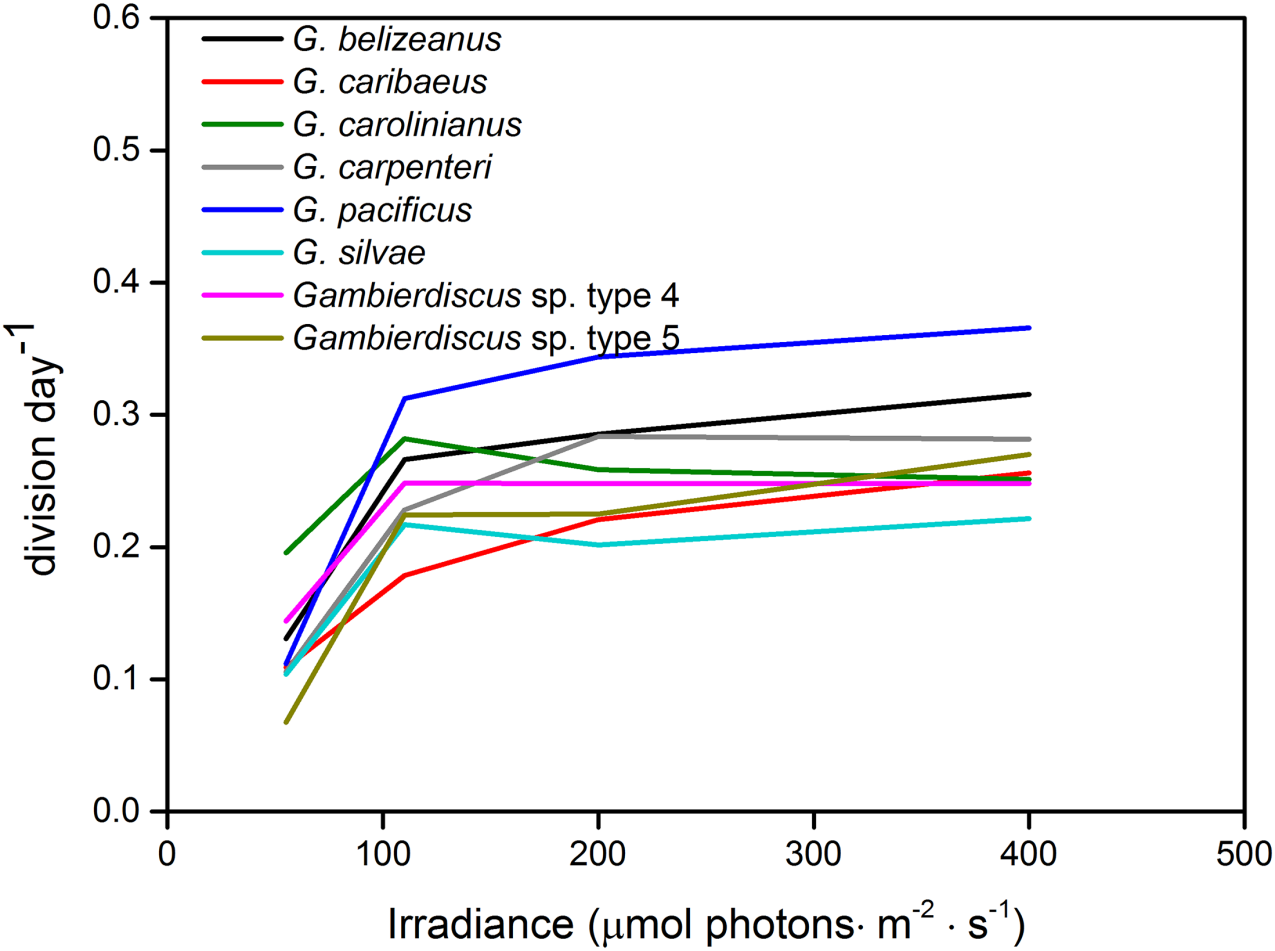
Average growth response of *Gambierdiscus* species to irradiance of 55–400μmol photons · m^-2^ · s^-1^. For ease of viewing, error bars shown in Fig 4 are omitted here.

Of the seven species/phylotypes examined, *G*. *pacificus* exhibited highest growth rates at irradiance levels ≥110μmol photons · m^-2^ · s^-1^ (Fig 5), and growth of both *G*. *pacificus* and *Gambierdiscus* sp. type 5 increased sharply when irradiance increased from 55 to 110μmol photons · m^-2^ · s^-1^ (Fig 5), suggesting low tolerance of low irradiances. In contrast, the slope of growth rate responses of *G*. *caribaeus* and *G*. *carolinianus* was comparatively flat (Fig 5), reflecting survival and growth at lower irradiance levels.

#### Intraspecific variation

CV values of *G*. *belizeanus*, *G*. *caribaeus*, *G*. *carolinianus*, *G*. *pacificus* and *Gambierdiscus* sp. type 4, varied from 0.5–89.9% (Table 4), and high CV values were typically seen at the lowest/highest irradiance levels (Table 4). Most intraspecific variability observed in the irradiance data was not statistically significant (α>0.05). However, for *G*. *belizeanus*, *G*. *caribaeus*, and *G*. *pacificus*, intraspecific growth rates were significantly different at the extreme irradiance levels of 55 or 400μmol photons · m^-2^ · s^-1^ (Table 4).

**Table 4:** *Gambierdiscus* strain numbers, growth rate, coefficient of variation among strains, and intraspecific difference for each species and irradiance (55–400μmol · photons · m^-2^ · s^-1^).

Species	Irradiance (μmol · photons · m^-2^ · s^-1^)	Number of strains	Growth rate (mean ± SD day^-1^)	Coefficient of variation (%)	p value
*G*. *belizeanus*	55	4	0.13 ± 0.01	5.8	0.564
*G*. *belizeanus*	110	4	0.27 ± 0.01	2.0	0.966
*G*. *belizeanus*	200	4	0.29 ± 0.02	6.9	0.134
*G*. *belizeanus*	400	4	0.32 ± 0.05	16.0	**0.001**
*G*. *caribaeus*	55	3	0.11 ± 0.03	25.7	**0.026**
*G*. *caribaeus*	110	3	0.18 ± 0.03	15.4	0.083
*G*. *caribaeus*	200	3	0.22 ± 0.02	6.8	0.607
*G*. *caribaeus*	400	3	0.26 ± 0.02	7.9	0.330
*G*. *carolinianus*	55	3	0.20 ± 0.02	11.2	0.100
*G*. *carolinianus*	110	3	0.28 ± 0.03	11.6	0.340
*G*. *carolinianus*	200	3	0.26 ± 0.02	7.7	0.421
*G*. *carolinianus*	400	3	0.25 ± 0.00	1.0	0.995
*G*. *pacificus*	55	2	0.28 ± 0.18	64.6	**0.001**
*G*. *pacificus*	110	2	0.33 ± 0.01	4.4	0.447
*G*. *pacificus*	200	2	0.32 ± 0.03	9.4	0.960
*G*. *pacificus*	400	2	0.20 ± 0.18	89.9	0.252
*Gambierdiscus* sp. type 4	55	2	0.14 ± 0.02	12.4	0.544
*Gambierdiscus* sp. type 4	110	2	0.25 ± 0.02	6.7	0.454
*Gambierdiscus* sp. type 4	200	2	0.25 ± 0.02	9.5	0.267
*Gambierdiscus* sp. type 4	400	2	0.25 ± 0.00	0.5	0.955

Irradiances at which intraspecific variation was significant are listed in bold (α< 0.05).

### Temperature

#### Growth rate

**Fig 6:**
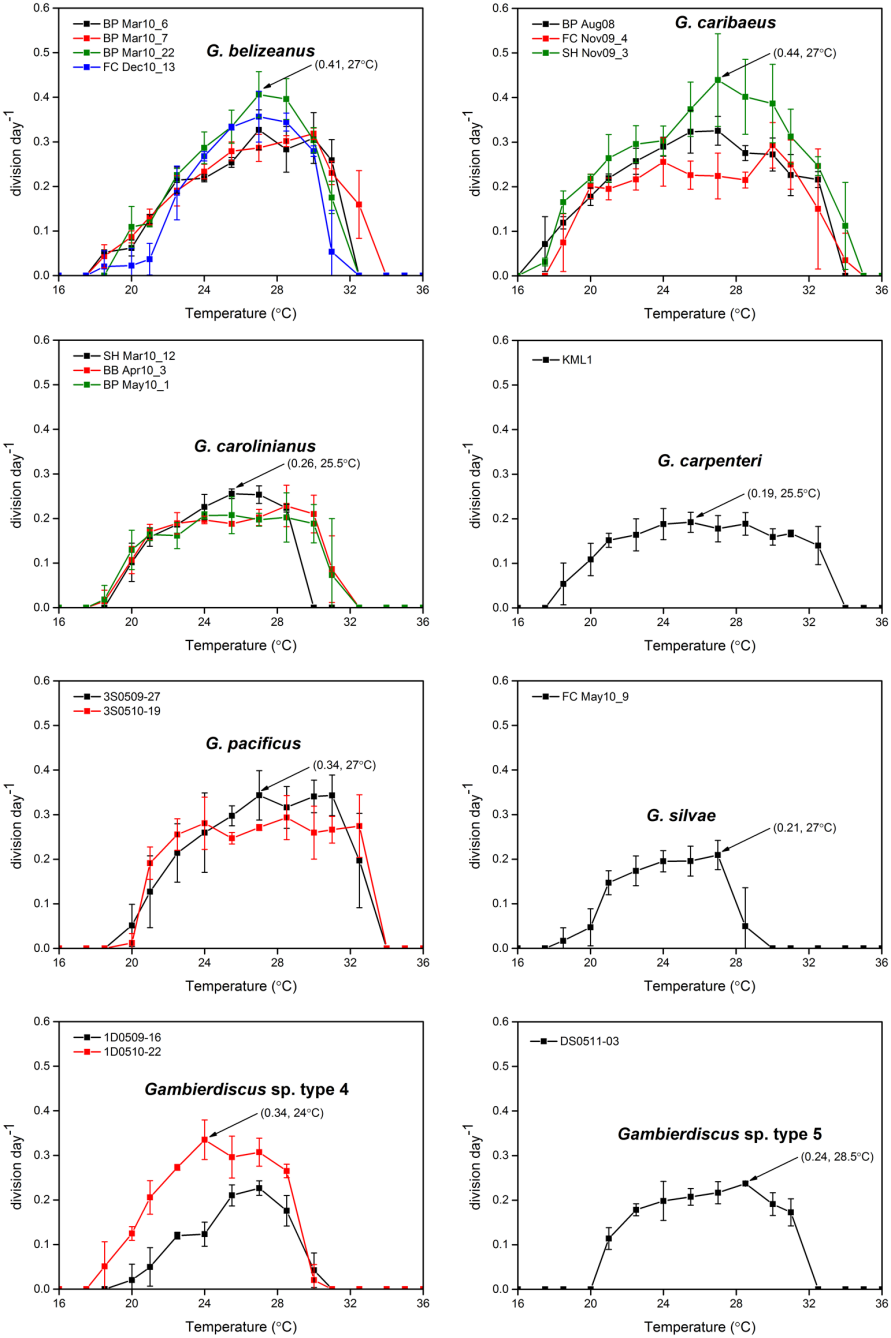
Growth rates of *Gambierdiscus* strains versus culture temperature of 16–36°C. Each point is the mean of triplicate measurements, and the bars represent standard deviation (SD). Black arrows represent the maximum growth rate for a species.

**Fig 7:**
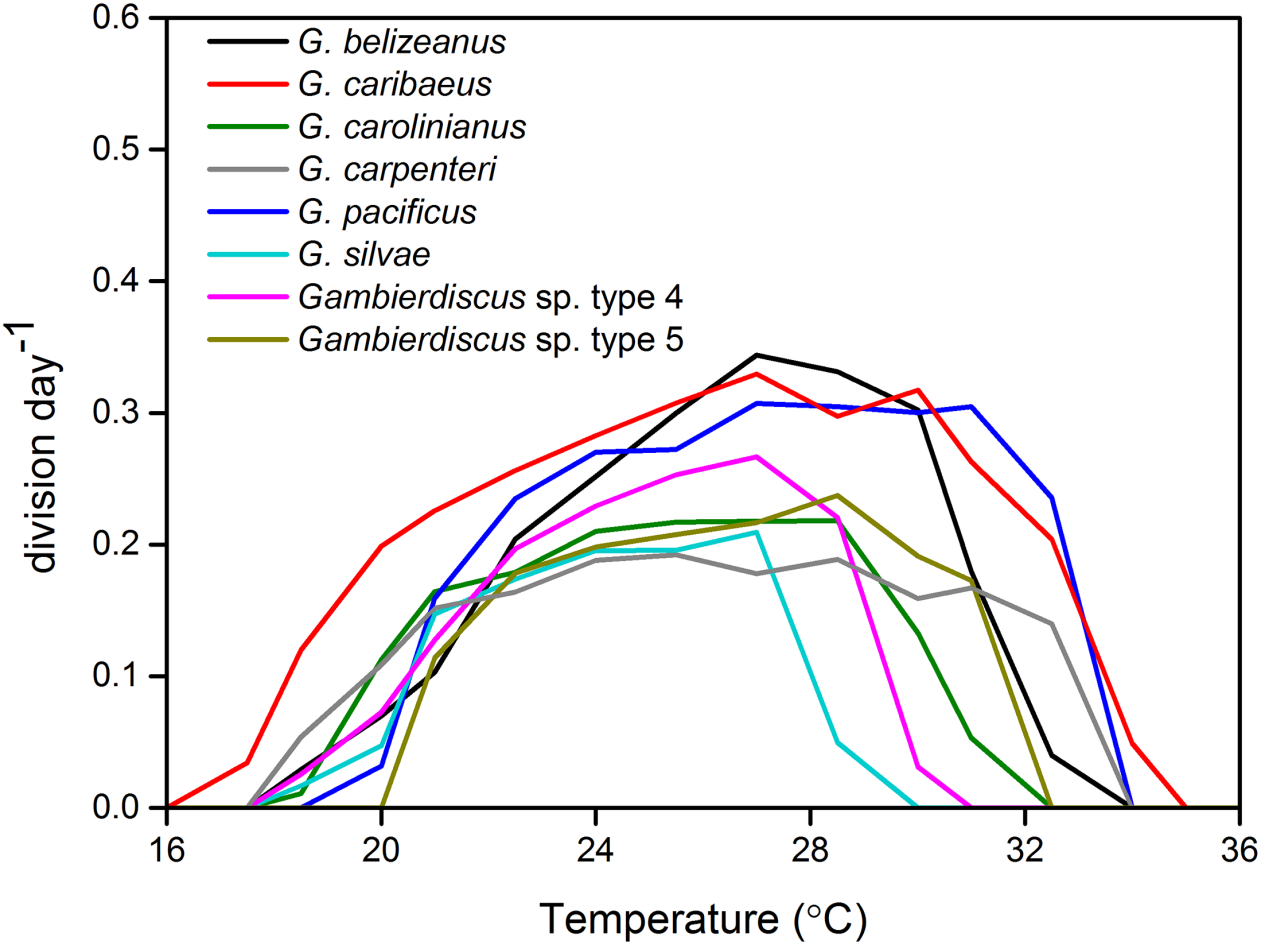
Average growth response of *Gambierdiscus* species to temperatures of 16–36°C. For ease of viewing, error bars shown in Fig 6 are omitted here.

As indicated in Figs 6 and 7, *Gambierdiscus* species responded to increasing temperatures in a Gaussian mode with enhanced growth to a threshold where rates were maximum, followed by plateau and then decrease in growth.

*Gambierdiscus* cells generally did not survive at temperatures ≤17.5°C and ≥ 32.5°C, although some strains exhibited a narrower range of temperature tolerance (Fig 6). For example, strains 3S0509-27 and 3S0510-19 (*G*. *pacificus*) stopped growing at temperatures ≤ 18.5°C, and strains of 1D0509-16 and 1D0510-22 (*Gambierdiscus* sp. type 4) died when temperatures exceeded 31°C (Fig 6). *Gambierdiscus* spp. appeared to be more sensitive to the higher extreme temperatures than the lower extreme temperatures; growth rates dropped dramatically when temperature approached the upper temperature limit, showing a steep decline in growth (Figs 6 and 7). In particular, *G*. *silvae* and *Gambierdiscus* sp. type 4 exhibited greatest sensitivity to high temperatures, with growth rates declining markedly between 28–30°C. Vegetative cells were generally present in cultures near the lower temperature limit, and were able to survive for periods of 25 days or more under these conditions, albeit with much reduced growth.

Overall, *Gambierdiscus* growth rates as a function of temperature varied from no growth to a maximum of 0.44 division day^-1^. For each species, the maximum growth rate observed at a given temperature was strain-dependent (Fig 6). Mean growth rates at the species level indicated that *G*. *belizeanus*, *G*. *caribaeus*, and *G*. *pacificus* had higher growth rates than the other species; furthermore, *G*. *caribaeus* and *G*. *carpenteri* were most tolerant to lower temperatures, and *G*. *caribaeus*, *G*. *carpenteri*, and *G*. *pacificus* were most tolerant to higher temperatures (Fig 7).

#### Intraspecific variation

The coefficient of variation (CV) was used to assess growth rate variability within species at each temperature (Table 5). For *G*. *belizeanus*, *G*. *caribaeus*, *G*. *carolinianus*, *G*. *pacificus*, and *Gambierdiscus* sp. type 4, the CV ranged from 3.5–200% across the temperature ranges analyzed (18.5–32.5, 17.5–34.0, 18.5–31.0, 20.0–32.5 and 18.5–30.0°C). Highest variability was reported at temperatures at the upper and lower limits, but *Gambierdiscus* spp. tested exhibited significant intraspecific variation as well (α<0.05), mainly at mid-range experimental temperatures (Table 5).

**Table 5:** *Gambierdiscus* strain numbers, growth rate, coefficient of variation among strains, and intraspecific differences for each species and temperature (16–38°C).

Species	Temperature (°C)	Number of strains	Growth rate (mean ± SD day^-1^)	Coefficient of variation (%)	p value
*G*. *belizeanus*	16.0	4	no growth		
*G*. *belizeanus*	17.5	4	no growth		
*G*. *belizeanus*	18.5	4	0.03 ± 0.02	81.2	0.227
*G*. *belizeanus*	20.0	4	0.07 ± 0.04	53.1	0.051
*G*. *belizeanus*	21.0	4	0.10 ± 0.04	43.2	**0.002**
*G*. *belizeanus*	22.5	4	0.20 ± 0.02	9.4	0.553
*G*. *belizeanus*	24.0	4	0.25 ± 0.03	12.4	**0.013**
*G*. *belizeanus*	25.5	4	0.30 ± 0.04	13.4	**0.005**
*G*. *belizeanus*	27.0	4	0.34 ± 0.05	14.6	0.072
*G*. *belizeanus*	28.5	4	0.33 ± 0.05	15.1	**0.022**
*G*. *belizeanus*	30.0	4	0.30 ± 0.17	5.5	0.107
*G*. *belizeanus*	31.0	4	0.18 ± 0.09	50.6	**0.010**
*G*. *belizeanus*	32.5	4	0.04 ± 0.08	200.0	**0.013**
*G*. *belizeanus*	34.0	4	no growth		
*G*. *belizeanus*	35.0	4	no growth		
*G*. *belizeanus*	36.0	4	no growth		
*G*. *belizeanus*	38.0	4	no growth		
*G*. *caribaeus*	16.0	3	no growth		
*G*. *caribaeus*	17.5	3	0.03 ± 0.04	104.5	**0.046**
*G*. *caribaeus*	18.5	3	0.12 ± 0.05	37.9	0.125
*G*. *caribaeus*	20.0	3	0.20 ± 0.02	10.2	0.123
*G*. *caribaeus*	21.0	3	0.23 ± 0.03	15.4	0.276
*G*. *caribaeus*	22.5	3	0.26 ± 0.04	15.5	0.063
*G*. *caribaeus*	24.0	3	0.28 ± 0.02	8.7	0.365
*G*. *caribaeus*	25.5	3	0.31 ± 0.08	24.4	**0.025**
*G*. *caribaeus*	27.0	3	0.33 ± 0.11	32.7	**0.025**
*G*. *caribaeus*	28.5	3	0.30 ± 0.10	32.0	**0.026**
*G*. *caribaeus*	30.0	3	0.32 ± 0.06	19.2	0.135
*G*. *caribaeus*	31.0	3	0.26 ± 0.04	17.0	0.213
*G*. *caribaeus*	32.5	3	0.20 ± 0.05	24.1	0.268
*G*. *caribaeus*	34.0	3	0.05 ± 0.06	116.8	0.189
*G*. *caribaeus*	35.0	3	no growth		
*G*. *caribaeus*	36.0	3	no growth		
*G*. *caribaeus*	38.0	3	no growth		
*G*. *carolinianus*	16.0	3	no growth		
*G*. *carolinianus*	17.5	3	no growth		
*G*. *carolinianus*	18.5	3	0.01 ± 0.01	88.6	0.558
*G*. *carolinianus*	20.0	3	0.11 ± 0.01	13.1	0.680
*G*. *carolinianus*	21.0	3	0.16 ± 0.01	3.5	0.740
*G*. *carolinianus*	22.5	3	0.18 ± 0.02	8.5	0.310
*G*. *carolinianus*	24.0	3	0.21 ± 0.01	7.0	0.205
*G*. *carolinianus*	25.5	3	0.22 ± 0.03	16.0	**0.006**
*G*. *carolinianus*	27.0	3	0.22 ± 0.03	14.3	**0.014**
*G*. *carolinianus*	28.5	3	0.22 ± 0.01	6.3	0.736
*G*. *carolinianus*	30.0	3	0.13 ± 0.12	87.0	**0.001**
*G*. *carolinianus*	31.0	3	0.05 ± 0.05	87.5	0.350
*G*. *carolinianus*	32.5	3	no growth		
*G*. *carolinianus*	34.0	3	no growth		
*G*. *carolinianus*	35.0	3	no growth		
*G*. *carolinianus*	36.0	3	no growth		
*G*. *carolinianus*	38.0	3	no growth		
*G*. *pacificus*	16.0	2	no growth		
*G*. *pacificus*	17.5	2	no growth		
*G*. *pacificus*	18.5	2	no growth		
*G*. *pacificus*	20.0	2	0.03 ± 0.03	87.3	0.258
*G*. *pacificus*	21.0	2	0.16 ± 0.05	28.5	0.277
*G*. *pacificus*	22.5	2	0.23 ± 0.03	12.5	0.390
*G*. *pacificus*	24.0	2	0.27 ± 0.01	5.5	0.750
*G*. *pacificus*	25.5	2	0.27 ± 0.04	13.1	**0.028**
*G*. *pacificus*	27.0	2	0.31 ± 0.05	16.6	0.150
*G*. *pacificus*	28.5	2	0.30 ± 0.02	5.3	0.592
*G*. *pacificus*	30.0	2	0.30 ± 0.06	19.1	0.114
*G*. *pacificus*	31.0	2	0.30 ± 0.05	17.9	0.071
*G*. *pacificus*	32.5	2	0.24 ± 0.05	23.1	0.353
*G*. *pacificus*	34.0	2	no growth		
*G*. *pacificus*	35.0	2	no growth		
*G*. *pacificus*	36.0	2	no growth		
*G*. *pacificus*	38.0	2	no growth		
*Gambierdiscus* sp. type 4	16.0	2	no growth		
*Gambierdiscus* sp. type 4	17.5	2	no growth		
*Gambierdiscus* sp. type 4	18.5	2	0.03 ± 0.04	141.4	0.185
*Gambierdiscus* sp. type 4	20.0	2	0.07 ± 0.07	101.5	**0.010**
*Gambierdiscus* sp. type 4	21.0	2	0.13 ± 0.11	86.3	**0.009**
*Gambierdiscus* sp. type 4	22.5	2	0.20 ± 0.11	55.1	**0.001**
*Gambierdiscus* sp. type 4	24.0	2	0.23 ± 0.15	65.4	**0.002**
*Gambierdiscus* sp. type 4	25.5	2	0.25 ± 0.06	24.0	**0.048**
*Gambierdiscus* sp. type 4	27.0	2	0.27 ± 0.06	21.3	**0.017**
*Gambierdiscus* sp. type 4	28.5	2	0.22 ± 0.06	28.7	**0.014**
*Gambierdiscus* sp. type 4	30.0	2	0.03 ± 0.02	50.0	0.504
*Gambierdiscus* sp. type 4	31.0	2	no growth		
*Gambierdiscus* sp. type 4	32.5	2	no growth		
*Gambierdiscus* sp. type 4	34.0	2	no growth		
*Gambierdiscus* sp. type 4	35.0	2	no growth		
*Gambierdiscus* sp. type 4	36.0	2	no growth		
*Gambierdiscus* sp. type 4	38.0	2	no growth		

Temperatures at which intraspecific variation was significant are listed in bold (α < 0.05).

#### Polynomial regression analysis

**Fig 8:**
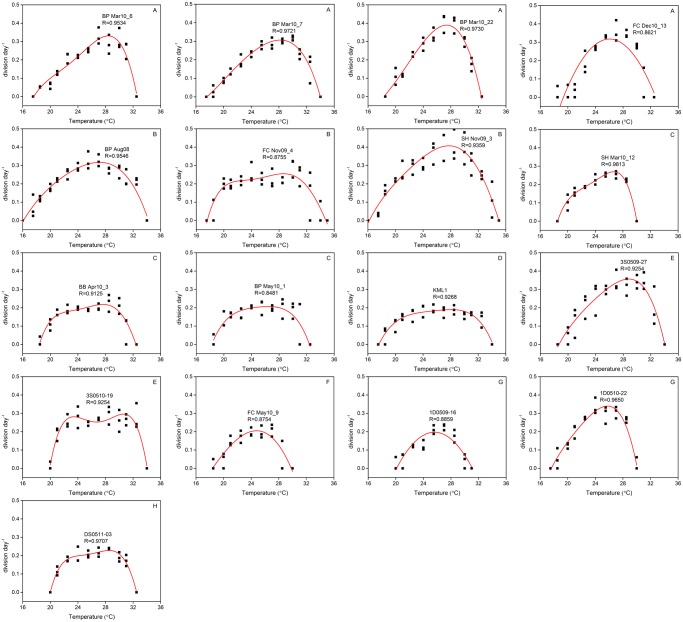
*Gambierdiscus* growth rate responses (black squares) at 16–36°C and simulated growth curves (red lines) by polynomial regressive analysis. (A) *G*. *belizeanus* (BP Mar10_6, BP Mar10_7, BP Mar10_22, FC Dec10_13), (B) *G*. *caribaeus* (BP Aug08, FC Nov09_4, SH Nov09_3), (C) *G*. *carolinianus* (SH Mar10_12, BB Apr10_3, BP May10_1), (D) *G*. *carpenteri* (KML1), (E) *G*. *pacificus* (3S0509-27, 3S0510-19), (F) *G*. *silvae* (FC May10_9), (G) *Gambierdiscus* sp. type 4 (1D0509-16, 1D0510-22), and (H) *Gambierdiscus* sp. type 5 (DS0511-03).

To evaluate *Gambierdiscus* growth potential at each temperature, growth rate and temperature were described using the polynomial equation: Y = A + B_1_X + B_2_X^2^ + … + B_n_X^n^ (n≤5) where X and Y represent temperature and growth rate, respectively. Major strains were fitted to a 4^th^ to 5^th^ order polynomial equation with an R value >0.85 (Fig 8, S2 Table).

In contrast to the polynomial curves determined for the salinity experiments, polynomial curves for temperature experiments could be divided into two types. One was nearly symmetrical, and included strains FC Nov09_4 (*G*. *caribaeus*), BB Apr10_3 and BP May10_1 (*G*. *carolinianus*), KML1 (*G*. *carpenteri*), 3S0510-19 (*G*. *pacificus*), FC May10_9 (*G*. *silvae*), 1D0509-16 (*Gambierdiscus* sp. type 4), and DS0511-03 (*Gambierdiscus* sp. type 5). In this group, major strains showed a consistent growth plateau over a wide temperature range, e.g., KML1 displayed stable, maximal growth over the temperature range of 21–28.5°C (Fig 8). The second growth type observed, where the cultures responded incrementally to temperature change and featured a skewed growth curve, included strains BP Mar10_6, BP Mar10_7, BP Mar10_22, and FC Dec10_13 (*G*. *belizeanus*), BP Aug08 and SH Nov09_3 (*G*. *caribaeus*), SH Mar10_12 (*G*. *carolinianus*), 3S0509-27 (*G*. *pacificus*), and 1D0510-22 (*Gambierdiscus* sp. type 4) (Fig 8).

*Gambierdiscus* growth parameters of *μ*_*m*_, *T*_m_, *T*_opt_, *T*_o_, and *T*_u_ differed among species, and within individual species; however, strains exhibiting skewed growth curves generally had higher *μ*_*m*_ and *T*_m_, narrower *T*_opt_ range, and lower *T*_o_ compared with strains with symmetrical growth curve (Table 6). Among the 17 strains tested, *μ*_*m*_ ranged from 0.14 to 0.41 division day^-1^, with 0.14 division day^-1^ measured in BP May10_1 (*G*. *carolinianus*) showing a symmetrical *μ*-°C shape, and 0.41 division day^-1^ in BP Mar10_7 (*G*. *belizeanus*) exhibiting a skewed *μ*-°C shape.

**Table 6:** *Gambierdiscus* species growth parameters in response to temperature (16–38°C). Individual growth rate measurements were fitted to polynomial curves and equations. The polynomial equations were used for growth parameter estimation: *μ*_*m*_, maximum growth rate; *T*_m_, temperature of maximum growth; *T*_opt_, temperature of optimum growth range (*μ*≥0.8×*μ*_m_); *T*_o_, the lower temperature limit for growth; *T*_u_, the upper temperature limit for growth.

Strain	Species	*μ*_*max*_	*T*_m_	*T*_opt_	*T*_opt_ range	*T*_o_	*T*_u_	*μ*-°C shape
BP Mar10_6	*G*. *belizeanus*	0.26	28.1	25.2–30.1	4.9	17.9	32.0	Skew
BP Mar10_7	*G*. *belizeanus*	0.41	29.1	24.9–32.3	7.4	16.8	35.2	Skew
BP Mar10_22	*G*. *belizeanus*	0.39	27.4	24.4–29.9	5.5	18.3	32.4	Skew
FC Dec10_13	*G*. *belizeanus*	0.32	26.1	23.1–29.1	6.0	19.3	32.8	Skew
BP Aug08	*G*. *caribaeus*	0.39	28.1	23.1–31.8	8.7	15.9	35.4	Skew
FC Nov09_4	*G*. *caribaeus*	0.22	28.1	20.1–31.1	11.0	17.6	34.0	Symmetrical
SH Nov09_3	*G*. *caribaeus*	0.33	26.9	22.8–30.1	7.3	16.4	33.6	Skew
SH Mar10_12	*G*. *carolinianus*	0.31	26.9	23.9–28.6	4.7	18.4	30.3	Skew
BB Apr10_3	*G*. *carolinianus*	0.17	27.0	20.8–29.4	8.6	18.6	31.6	Symmetrical
BP May10_1	*G*. *carolinianus*	0.14	23.8	20.9–27.9	7.0	18.5	30.9	Symmetrical
KML1	*G*. *carpenteri*	0.24	29.2	22.7–32.3	9.6	17.5	35.0	Symmetrical
3S0509-27	*G*. *pacificus*	0.34	28.7	25.0–31.3	6.3	18.8	33.9	Skew
3S0510-19	*G*. *pacificus*	0.30	26.2	22.4–32.8	10.4	19.8	34.5	Symmetrical
FC May10_9	*G*. *silvae*	0.20	24.8	22.2–27.1	4.9	18.4	29.8	Symmetrical
1D0509-16	*Gambierdiscus* sp. type 4	0.20	25.6	23.1–28.1	5.0	20.1	31.1	Symmetrical
1D0510-22	*Gambierdiscus* sp. type 4	0.33	25.7	22.8–27.9	5.1	17.6	30.0	Skew
DS0511-03	*Gambierdiscus* sp. type 5	0.17	27.9	22.0–30.1	8.1	20.1	31.9	Symmetrical

*Gambierdiscus carpenteri* required the highest *T*_m_ to realize maximum growth, in comparison with *G*. *silvae*, which required the lowest *T*_m_ (Table 6). In addition, species of *G*. *belizeanus*, *G*. *caribaeus*, and *G*. *pacificus* generally exhibited a high *μ*_*m*_, versus *Gambierdiscus* type 5, which exhibited a low *μ*_*m*_ (Table 6). Furthermore, species of *G*. *caribaeus*, *G*. *carpenteri*, and *G*. *pacificus* typically had a wide range of *T*_opt_; *G*. *caribaeus*, and *G*. *carpenteri* displayed a low *T*_o_; and *G*. *belizeanus*, *G*. *caribaeus*, *G*. *carpenteri*, and *G*. *pacificus* displayed a high *T*_u_ (Table 6). These species thus tolerated extreme temperatures better than others. In contrast, species of *G*. *silvae* and *Gambierdiscus* types 4–5 were sensitive to extreme temperature, exhibiting either narrow *T*_opt_, or high *T*_o_, or low *T*_u_ (Table 6).

## Discussion

This study examined the growth response patterns of multiple strains of eight *Gambierdiscus* species/phylotypes under different salinity, irradiance, and temperature, including the first characterization of *G*. *silvae* and *Gambierdiscus* sp. type 4–5. All strains used were isolated from geographically distinct areas compared with strains used in previous studies. The results showed that environmental variability in salinity, irradiance, and temperature can greatly influence *Gambierdiscus* growth, which was revealed by both intraspecific and interspecific variation. In general, strains of *G*. *belizeanus*, *G*. *caribaeus*, *G*. *carpenteri*, and *G*. *pacificus* exhibited a wider range of tolerance to extreme environmental conditions than the other species, consistent with their broad geographic distribution. The growth response of *Gambierdiscus* to environmental parameters is clearly a major determinant of the species’ abundance and distribution in natural ecosystems, and is useful in evaluating and understanding current and future species distributions and population dynamics both within systems and across geography (e.g. [5]).

### Growth rates

Compared with planktonic dinoflagellates, the epibenthic genus *Gambierdiscus* is slow-growing. Maximum growth rates are generally lower than 0.5 division day^-1^ [35] and growth rates of approximately 0.3 division day^-1^ are commonly observed [36], consistent with this study. Thus far, the highest *Gambierdiscus* growth rate reported is 0.79 division day^-1^ (0.55/day) for a Hawaiian strain [37], and under optimum combinations of temperature, salinity and light, a growth rate of >0.5 division day^-1^ was also possible for a Florida strain, GT600 [3]. Unfortunately, the species used in these studies are unknown.

Growth responses of *G*. *belizeanus*, *G*. *caribaeus*, *G*. *carolinianus*, and *G*. *pacificus* analyzed during this study were similar to previous reports; however, *G*. *carpenteri* exhibited a narrower range of growth than in previous studies (see comparison in Table 7). Most of these reported growth rates were measured over a range of optimal growth regimes of salinity, irradiance, and temperature (Table 7). Kibler et al. [29] measured growth at 15–34°C, salinity of 33, and 50–100μmol photons·m^-2^·s^-1^, while Yoshimatsu et al. [7] measured at 15–35°C, salinity of 20–40, and 90–100μmol photons·m^-2^·s^-1^. Disparities in growth rates in these experiments most likely reflect genetic differences in growth response rather than culture conditions.

**Table 7:** Comparison of *Gambierdiscus* species growth rates between the current and previous studies.

Species	This study		Other studies	
	Strain Number	Growth rate (division day ^-1^)	Strain Number	Growth rate (division day ^-1^)
*G*. *belizeanus*	4	0–0.41	1	0.14 [22]
			1	0 - ~0.29 [28]
			2	0 - ~0.35 [29]
*G*. *caribaeus*	3	0–0.44	1	~0.10–0.20 [21]
			1	0–0.24 [23]
			1	0 - ~0.48 [28]
			NA	~ 0.29 [30]
			6	0 - ~0.46 [29]
*G*. *carolinianus*	3	0–0.48	1	0 - ~0.46 [28]
			5	0 - ~ 0.51 [29]
*G*. *carpenteri*	1	0–0.29	1	0 - ~0.55 [5]
*G*. *pacificus*	2	0–0.42	2	0.18–0.21 [22]
			1	0 - ~0.29 [28]
			1	0 - ~0.36 [5]
*G*. *silvae*	1	0–0.21		NA
*Gambierdiscus*. sp. type 4	2	0–0.34		NA
*Gambierdiscus* sp. type 5	1	0–0.27		NA
*G*. *australes*		NA	5	0.12–0.19 [22]
			1	0 - ~0.43 [5]
			1	0–0.26 [7]
*G*. *polynesiensis*		NA	3	0.13–0.17 [22]
*G*. *ruetzleri*/ *F*. *ruetzleri*		NA	1	0 - ~0.50 [28]
			2	0 - ~0.53 [29]
*G*. *scabrosus*		NA	1	0–0.40 [7]
*G*. *toxicus*		NA	5	0.16–0.19 [22]
*Gambierdiscus* sp. ribotype 2		NA	1	~0.14–0.29 [21]
			1	0 - ~0.17 [28]
			7	0 - ~0.28 [29]
*Gambierdiscus* sp. type 2		NA	1	0–0.24 [7]
*Gambierdiscus* sp. type 3		NA	1	0–0.37 [7]

NA: Not available

Growth rates for [28], [5] and [29] were estimated from Fig 5 in Tester et al. [28], Figs 2–4 in Kibler et al. [5] and Fig 4 in Kibler et al. [29], respectively. Data in day^-1^ were converted into data in division day^-1^ referring to Guillard [33].

Additionally, growth rates of *Gambierdiscus* determined in this study varied among species, and within individual species (Table 7), even for strains that were isolated from the same location in the same survey (e.g., 1D0509-16 and 1D0510-22). These findings contrast with observations by Bomber et al. [26], who found no significant difference in growth rates among strains from the same station, and concluded that one strain per site was probably representative. Our findings are more similar to results reported by Boyd et al. [38] and Burkholder [39], who demonstrated that it is misleading to use a single strain to represent a phytoplankton functional group.

The aforementioned *Gambierdiscus* growth pattens add complexity to ciguatera prediction and its management, particularly with respect to model development. Currently it is unknown whether *Gambierdiscus* toxin production is strain-dependent, but it certainly seems likely. If *Gambierdiscus* toxin production is stable within species, ciguatera monitoring efforts may be best focused on toxic species with high growth rates. However, if *Gambierdiscus* toxicity is strain-dependent, as we expect, developing an effective ciguatera monitoring and prediction program will be more difficult. Further efforts are currently underway to determine the species and strain variability of toxin production in *Gambierdiscus*.

### Salinity

#### Growth response to salinity

*Gambierdiscus* growth responses to varying salinity (10–60) were nonlinear with an approximate Gaussian/bell shape (Fig 3). Compared with temperature, the growth curves for salinity were more symmetrical, indicating that *Gambierdiscus* cells are less sensitive to hypersaline conditions than they are to high temperatures. This is supported by observations by Yoshimatsu et al. [7] that the effect of temperature on growth of Japanese *Gambierdiscus* was stronger than those of salinity or temperature-salinity varying together.

The growth responses to varying salinity described in this study are similar in nature to previous reports, but the salinities at which optimal growth and growth inhibition occurred were markedly different [3–5, 7]. For example, in a laboratory unialgal culture, little growth was observed at a salinity of 45 [3]; similarly, clone GT600A could not survive in salinities >43 [4], and growth of Japanese *Gambierdiscus* spp. was not supported at salinity levels above 40 [7]. These reports differ from our observations, and those reported by Kibler et al. [5]. Comparing parameters of *S*_m_, *S*_o_, and *S*_u_ estimates in the present study with those in Kibler et al. [5], i.e., 30–39 vs. 25–35, 10–27 vs. <14–21, and 48–57 vs. 39->41, respectively, strains from this study required higher salinities to realize maximal growth, and were less sensitive to hypo/hyper salinity. Differences between these two studies may be due to both intra- and inter-specific variability.

Intraspecific variability in the salinity experiments was similar to those observed in temperature experiments, i.e., *Gambierdiscus* cells exhibited larger CV values near either end of the salinity range than in the middle. Growth potential appears to be significantly different within species, especially in the vicinity of optimum salinity (Table 2), which provides further evidence of intra-specific physiological diversity. According to Boyd et al. [38], the distribution and expansion of an organism in neritic waters largely depends upon intraspecific variability in response to temperature. Similarly, the intraspecific variability in growth responses to salinity may help explain why *Gambierdiscus* spp. are widely distributed in the tropical, subtropical, and temperate regions. In addition to temperature tolerance, the growth response of species of *G*. *belizeanus*, *G*. *caribaeus*, and *G*. *carpenteri* to different salinities under laboratory conditions provides physiological evidence as to why these species are widely distributed [20].

#### Salinity and *Gambierdiscus* abundance

*Gambierdiscus* generally prefers high, stable salinities of 28–35 (summarized by [6]), though this estimate was recently updated to include a broader range than was previously reported [5, 7]. Salinities of 34–38 are typical for oceanic waters in areas with ciguatera, thus oceanic salinity should sustain maximum growth of most *Gambierdiscus* species. This is supported by field observations; for example, no relationship between *Gambierdiscus* abundance and water salinity was found in French Polynesia, where salinities ranged from 34.3–36.1 [1], which was optimal for *Gambierdiscus* bloom formation. At the Flower Garden Banks National Marine Sanctuary in the northern Gulf of Mexico, high biodiversity of *Gambierdiscus* was observed (i.e., six of the seven *Gambierdiscus* species endemic to the Caribbean region); again, salinity levels of 34–37 measured during the survey support optimum growth [40].

In contrast with the stable or narrow range of salinity in oceanic regions, some coastal locations such as estuaries and bays are affected by freshwater inputs from precipitation and freshwater discharge from land. At these locations, hyposaline conditions may inhibit *Gambierdiscus* survival. For example, below a salinity of 14 in the coastal zone in the Gulf of Mexico [41], only *G*. *caribaeus* and *G*. *carpenteri* have a good chance of survival or growth.

Besides hyposaline conditions mentioned, hypersaline environments pose another challenge for *Gambierdiscus* growth. These conditions readily arise in restricted water bodies in the tropical and subtropical areas with high evaporation, poor circulation, and low freshwater input such as tropical lagoons, where salinities can easily exceed 40 [42]. In response to hypersaline pressure, only *Gambierdiscus* isolates from *G*. *belizeanus*, *G*. *caribaeus*, and *G*. *carpenteri* may be able to grow.

In previous ecological surveys, positive or negative correlations between *Gambierdiscus* and salinity were observed only under extreme salinity conditions (hyposaline and hypersaline). For example, *Gambierdiscus* was absent from river mouth sites due to low salinity [25, 43]. Another widely known observation comes from the Virgin Islands; regional precipitation (lower salinity) was significantly and positively related with *Gambierdiscus* abundance at inshore stations, with *Gambierdiscus* population maxima co-occurring with peak rainfall [44]. The apparent paradox between these two reports may be attributed to the different ways precipitation influences *Gambierdiscus* populations. Freshwater inputs from precipitation could be advantageous due to nutrient inputs if water salinity remains within a suitable range for growth, but could be disadvantageous when hyposalinity conditions occur that are suboptimal for *Gambierdiscus* growth.

Within the context of climate change, precipitation patterns are predicted to occur in which rainfall is less frequent but more intense, and followed by longer dry periods [45]. This new pattern is expected to favor dinoflagellate growth due to increases in water stratification and the availability of nutrients for growth [45, 46]. It is uncertain how *Gambierdiscus* will respond; as benthic organisms, they are distinct from the phytoplanktonic dinoflagellates in that they are generally associated with a benthic macroalgal habitat. Furthermore, one previous salinity shock experiment revealed that growth responses of *Gambierdiscus* to instantaneous salinity decreases were species-dependent and included a range of responses such as no effect, slowed growth, or mortality [5]. Further work that includes additional *Gambierdiscus* species and geographically distinct strains is thus required.

### Irradiance

Here, all 17 *Gambierdiscus* strains tested grew at 55–400μmol photons · m^-2^ · s^-1^, and no obvious growth inhibition was observed at 400μmol photons · m^-2^ · s^-1^. This pattern contrasts with other laboratory studies examining the irradiance response of several *Gambierdiscus* species. Clones GT600 and GT600A have been shown to be inhibited at irradiances of >232 and >225μmol photons · m^-2^ · s^-1^, respectively (units conversion refers to [47]). Similarly, *G*. *caribaeus* growth decreased when irradiance exceeded 300μmol photons · m^-2^ · s^-1^, and *G*. *carolinianus* and *G*. *pacificus* could not survive at 200 and 400μmol photons · m^-2^ · s^-1^, respectively [5]. No obvious growth inhibition was observed in this study when *Gambierdiscus* cells were cultured at 110–400μmol photons · m^-2^ · s^-1^. As in the temperature and salinity experiments, intraspecific variability may play a crucial role in explaining the differences among observations. In Kibler et al. [5], all eight *Gambierdiscus* species exhibited low light adaptation, requiring only 6–17μmol photons · m^-2^ · s^-1^ to maintain growth. Here, under the lowest irradiance tested, 55μmol photons · m^-2^ · s^-1^, all eight *Gambierdiscus* species/phylotypes examined grew and no mortality was recorded; light tolerance of <55μmol photons · m^-2^ · s^-1^ is thus likely.

There is an apparent inconsistency between laboratory findings and field observations of *Gambierdiscus* response to light intensity. The genus *Gambierdiscus* typically attains optimum growth at ~10% of full sunlight [4] or maxima growth at ~2.5–10% of surface irradiance [5], which agrees well with the general irradiance requirement for dinoflagellates [48]. Interestingly, growth responses to irradiance observed in this study were diverse, which may help explain why data obtained experimentally does not always reflect field observations of *Gambierdiscus* in shallow environments subjected to high light intensities. This study determined that the optimum light intensity for growth was ~4.4–16% of full light (full sunlight = 2500μmol photons · m^-2^ · s^-1^), which is higher than reported previously. However, it does not follow ecological observations in which *Gambierdiscus* cells were detected in the shallow waters of 1–5 m [49], on sparse macroalgae and bright sand flats [3], on drifting seaweed [10, 26], or floating detritus [27]. The tolerance for high-irradiance environments may be partly attributed to the finely-branched and three-dimensional structure of host macroalgae, which provides substrate for *Gambierdiscus* attachment and shields cells from strong light damage [50]. This hypothesis was indirectly verified by PAM fluorescence in that *Gambierdiscus* spp. exhibited typical characteristics of “shade-adapted” organisms [51].

Another explanation is that like other benthic dinoflagellates such as in the genus *Ostreopsis* [52], *Gambierdiscus* cells tend to produce more mucus at higher irradiance levels. This mucus production causes cell aggregation and a cell complex enveloped by large quantities of mucus protects cells from high light due to self-shading. Besides the physical structures that *Gambierdiscus* spp. utilize to shade themselves from high irradiance, photoprotection mechanisms observed in other dinoflagellates such as secretion of UV radiation-absorbing compounds and changes in pigment composition should also be considered [53, 54].

Irradiance plays an important role in defining *Gambierdiscus* vertical distribution in the water columns [5]. In most prior field studies, the genus *Gambierdiscus* was largely collected from shallow water depths <5 m [49], but were also recorded at depths of 10–40 m [40, 55]. Thus far, the maximum depth reported was for *G*. *carolinianus*, which was collected from 45.7 m in the northern Gulf of Mexico [40]. These findings are supported by other studies indicating that irradiance levels of 6–17μmol photons · m^-2^ · s^-1^ are sufficient to maintain *Gambierdiscus* growth, corresponding to >150m depth in tropical waters [5]. Since both temperature and irradiance decrease with increasing water depth, *Gambierdiscus* spp. in deep waters are not likely to experience conditions for optimal growth; however, *Gambierdiscus* cells inhabiting these ecosystems may serve as source populations for surrounding shallower niches.

### Temperature

#### Growth response to temperature

Although a linear relationship has been observed between *Gambierdiscus* growth responses and some environmental parameters such as DIN and phosphate [23], the growth responses to temperature we observed were near Gaussian in shape, with some strains exhibiting a more symmetrical shape than others (Fig 8). This near Gaussian response, seen frequently in similar studies of other phytoplankton species, indicates that *Gambierdiscus* growth is optimal and suboptimal within discrete temperature ranges, which is consistent with many previous observations [3–5, 29]. For *G*. *caribaeus*, the symmetric or slightly skewed growth response curve observed in this study is similar to that in Tester et al. [28] and Kibler et al. [29]. However, this response is markedly different from *G*. *caribaeus* in Kibler et al. [5], which had a highly skewed growth rate shape in response to temperature. For *G*. *belizeanus*, *G*. *carolinianus*, and *G*. *pacificus*, growth response shapes reported here and those described by Kibler et al. [5, 29] are not identical but can be regarded as analogous.

Our findings, together with others [5, 23, 28, 29], suggest that the global distribution of *G*. *belizeanus*, *G*. *caribaeus*, and *G*. *carpenteri* may be due in part to their broad tolerance to environmental conditions, especially to temperature. It is not surprising that *G*. *pacificus* also exhibited a wide thermal tolerance, as that species is frequently observed and is broadly disturbed in the tropical Pacific. Regarding *G*. *carolinianus*, our data agree with previous work indicating that this species has a relatively low maximum temperature for growth (*T*_m_ = ~25–27.1°C) and is well-adapted to lower temperatures (*T*_o_ = 15.8°C) [28, 29]. This species has been isolated as far north as North Carolina on the USA east coast, near 34° N [9] and collected from the deepest site ever recorded (45.7 m) for the genus [40]. The temperature parameters *T*_m_ and *T*_u_ of *G*. *carolinianus* are similar to previous results: 23.8–27.0°C vs. ~25–27.1°C and 30.3–31.6°C vs. 32.5°C, respectively [28, 29]; however, *T*_o_ in the current study was much higher (18.4–18.6°C vs. 15.8°C) [29]. These differences suggest that *G*. *carolinianus* originating from St Thomas, USVI may be more sensitive to lower temperatures than the strain isolated from North Carolina. It also implies that differences among strains within each species may reflect geographic origin, and cautions against using one strain to represent the physiological characteristics of a species.

To our knowledge, this study represents the first characterization of the relationship between temperature and growth for *G*. *silvae* and *Gambierdiscus* types 4 and 5. To date, only *Gambierdiscus* sp. ribotype 2 and types 2–3 were used in experiments assessing growth response to temperature. Similar to the findings of this study, *Gambierdiscus* sp. ribotype 2 and type 3 had a narrow *T*_opt_, corresponding to 24.5–30.1 and 22–25°C, respectively [7, 29]. But *Gambierdiscus* sp. type 2 showed a *T*_opt_ of 21–28°C [7]. Subsequent work examining additional strains will determine if similar patterns of intra-specific diversity also exists within these ribotypes.

The majority of growth studies carried out on *Gambierdiscus* used a single strain to represent each species [5, 7, 28]; however, like Kibler et al. [29], we examined multiple strains per species. In the temperature experiments, we observed intraspecific variability, including the response shape, and all parameters - *μ*_m_, *T*_m_, *T*_opt_, *T*_o_, and *T*_u_. Although the intraspecific CV of growth rate varied most at either end of the temperature range, statistically significant intraspecific variation was encountered within the *T*_opt_ range. This agrees with Tindall and Morton [49], who noted that competitive growth rates of *Gambierdiscus* and other ciguatera associated dinoflagellates could only be compared near the temperature for optimal growth. Our results are also similar to those documented by Boyd et al. [38], who reported that CV values among strains in both diatoms and dinoflagellates are lowest near the optimal growth temperature, and highest at the extremes of temperature tolerance, suggesting strong genotypic selection pressure at these end points [38].

#### Temperature and *Gambierdiscus* distribution

Temperature plays an essential role in restricting the distribution of epiphytic dinoflagellates. The genus *Gambierdiscus* generally has an optimal temperature range of 19–31°C, and sustains growth between 15–34°C (this study, [5, 7, 29]). The optimum temperature for oceanic phytoplankton is considered to be strongly related to the mean environmental temperature that species are exposed to [56]; the high temperature requirement for *Gambierdiscus* growth thus explains why this organism and ciguatera incidence are circumtropically endemic.

The parameter *T*_o_ indicates the extent to which *Gambierdiscus* is adapted to low temperatures and helps to define its latitudinal distribution. The growth measurements of 17 *Gambierdiscus* strains in this study produced *T*_o_ estimates varying from 15.9–20.1°C, which are close to previous laboratory reports, such as 16–19.5°C for Florida Keys isolate GT600 [3] and 15.0–20.0°C for multiple species/strains [5, 29]. Field surveys and laboratory culturing, however, show that *Gambierdiscus* species can survive in a broad range of temperate environments. Along the Pacific coast in Japan, *Gambierdiscus* cells occurred year-round in locations where the wintertime temperatures drop to as low as ~11°C [31], which represents the lowest field temperature recorded thus far for the genus. Under laboratory conditions, *Gambierdiscus* cultures established from the temperate coasts in Japan survived for at least three months at 10°C [31].

Using the lowest *T*_o_, 15.9°C from BP Aug08 (*G*. *caribaeus*), and wintertime Sea Surface Temperature (SST) (http://www.nodc.noaa.gov/about/oceanclimate.html) to predict its possible distribution boundary, this *G*. *caribaeus* strain may extend as far north as ~38.0°N, and as far south as ~45.0°S. This predicted range is narrower than the one described in Kibler et al. [5], who concluded that the greatest latitudes of distribution are approximately 38.5°N, and 47.1°S. The difference between these two predictions of *Gambierdiscus* distribution is attributed to the different *T*_o_ values used; in Kibler et al. [5], *T*_o_ was 15°C (NOAA 6, *G*. *carolinianus*) versus 15.9°C from our study. Clearly, a 1.0°C difference in *T*_o_ results in a marked change in *Gambierdiscus*’ predicted latitudinal range. However, the boundaries predicted in this study or that of Kibler et al. [5] are still broader than the observed field distribution of *Gambierdiscus*; i.e. 35°N-37°S [6]. This suggests that continued field sampling may discover additional *Gambierdiscus* populations beyond this range.

The temperature parameter *T*_u_ is another important index for determining how *Gambierdiscus* responds to high temperature. Estimates of *T*_u_ fell within 29.8–35.4°C, a broad range that encompasses previous *T*_u_ records, such as 31.1–35.1°C and 25–30°C for multiple isolates in Kibler et al. [5, 29] and Yoshimatsu et al. [7], respectively. The summer oceanic SST is generally within the *T*_u_ range of *Gambierdiscus* (http://www.nodc.noaa.gov/about/oceanclimate.html), with the warmest areas primarily located in the tropical Caribbean Sea, and the Pacific and Indian Oceans. Within these regions, the Indo-Pacific Warm Pool (IPWP) and West Pacific Warm Pool (WPWP) are two large bodies of seawater whose SST consistently remains above 28.5°C [32]. If the temperatures of the IPWP and WPWP are below *T*_u_, *Gambierdiscus* populations are capable of growth and development, provided that other environmental conditions are suitable for growth. However, once conditions exceed *T*_u_ for specific *Gambierdiscus* species or strains, mortality may occur, possibly altering the composition of *Gambierdiscus* populations in the system. This may help explain why nations in close proximity to the IPWP have low or negligible ciguatera rates even though they are located within the tropics [28, 32], generally regarded as ideal habitat for *Gambierdiscus*. Similar conditions exist in Red Sea and Arabian Sea, where rates of ciguatera are also negligible [32]. *Gambierdiscus* cells have been observed in these areas [24, 57], where seawater temperatures sometimes reach as high as 35°C [32]. On the basis of laboratory results, SSTs at or above 35°C would be lethal to most *Gambierdiscus* strains.

*Gambierdiscus* abundance is thought to be closely related to ocean warming. If warming waters still satisfy their growth requirement, a positive correlation between temperature and abundance may be expected [23]. This highlights an important point on semantics; some studies (e.g., [28]) have used observations of increasing ciguatera incidence or increased *Gambierdiscus* growth rates with increasing temperatures to argue that there will be range extensions or expansions. Clearly, however, if temperatures exceed thermal tolerance thresholds, *Gambierdiscus* spp. abundance and ciguatera may decline or disappear in some areas [23, 32], such that the net effect is not necessarily a range expansion of a species, but rather a shift in that range.

The Intergovernomental Panel on Climate Change (IPCC) estimated that by 2100, ocean SSTs will increase by 0.6–2.0°C, with highest increase occuring in tropical and Northern Hemisphere subtropical regions [58]. Given that the temperature gap between *T*_opt_ and *T*_u_ varied from 1.7–3.7°C in this study and 1.2–3.3°C in Kibler et al. [5, 29], one may expect that ocean warming in the next 100 years will inhibit those *Gamberdiscus* strains with a narrow *T*_opt_-*T*_u_ gap (e.g., *G*. *carolinianus* and *G*. *pacificus*, Table 6). Additionally, strains with a relatively low *T*_u_ (e.g. BP Mar10_6 and SH Mar10_12, Table 6) may be inhibited by this warming. Ocean warming is expected to result in declining tropical phytoplankton diversity, as many tropical strains, in the absence of evolution, are unable to survive even small increases in temperature [56]. The epibenthic dinoflagellate *Gambierdiscus* is no exception due to its sensitivity to the upper temperature range (*T*_u_), and narrow gaps between *T*_opt_ and *T*_u_. However, seawater does not have an infinite capacity to warm, and a phenomena called the tropical thermostat may suppress ocean warming [59]. As a result, the future relationship between *Gambierdiscus* and ciguatera due to rising seawater temperatures is complex. Nevertheless, under conditions of warming, *Gambierdiscus* populations will likely proliferate in some areas, and possibly decrease in regions where ocean temperatures exceed *T*_u_.

## Supporting Information

S1 Table Growth rate statistics calculated with polynomial fit of *Gambierdiscus* growth rates in response to salinity (10–60). (DOC)

S2 Table Growth rate statistics calculated with polynomial fit of *Gambierdiscus* growth rates in response to temperature (16–38°C). (DOC)
