# Supplementary material for: Influence of Environmental Variables on Gambierdiscus spp. (Dinophyceae) Growth and Distribution
Source: PLoS One. 2016 Apr 13;11(4):e0153197. doi: 10.1371/journal.pone.0153197 (PMC4830584; doi:10.1371/journal.pone.0153197)
Supplement: S1 Table — (DOC) [file pone.0153197.s001.doc]

S1. Growth rate statistics calculated with polynomial fit of *Gambierdiscus* growth rates in response to salinity (10-60).

| **Strain** | **Species** | **polynomial order** | **Number of Points** | **DF** | ***RSS*** | **Adj. R-Square** | **R Value** |
| --- | --- | --- | --- | --- | --- | --- | --- |
| BP Mar10_6 | *G. belizeanus* | 3 | 30 | 26 | 0.0424 | 0.86321 | 0.93667 |
| BP Mar10_7 | *G. belizeanus* | 4 | 27 | 22 | 0.02672 | 0.91722 | 0.96434 |
| BP Mar10_22 | *G. belizeanus* | 5 | 24 | 18 | 0.01317 | 0.94142 | 0.97681 |
| FC Dec10_13 | *G. belizeanus* | 5 | 27 | 21 | 0.03136 | 0.90849 | 0.96234 |
| BP Aug08 | *G. caribaeus* | 5 | 30 | 24 | 0.02448 | 0.93292 | 0.97185 |
| FC Nov09_4 | *G. caribaeus* | 4 | 27 | 22 | 0.02743 | 0.86813 | 0.94256 |
| SH Nov09_3 | *G. caribaeus* | 3 | 30 | 26 | 0.08167 | 0.84445 | 0.92765 |
| SH Mar10_12 | *G. carolinianus* | 4 | 24 | 19 | 0.06421 | 0.91084 | 0.96247 |
| BB Apr10_3 | *G. carolinianus* | 3 | 24 | 20 | 0.05186 | 0.82413 | 0.92036 |
| BP May10_1 | *G. carolinianus* | 5 | 21 | 15 | 0.00971 | 0.93748 | 0.97627 |
| KML1 | *G. carpenteri* | 4 | 27 | 22 | 0.05229 | 0.81769 | 0.91964 |
| 3S0509-27 | *G. pacificus* | 5 | 27 | 21 | 0.04676 | 0.90076 | 0.95908 |
| 3S0510-19 | *G. pacificus* | 4 | 27 | 22 | 0.04871 | 0.90116 | 0.95727 |
| FC May10_9 | *G. silvae* | 2 | 18 | 15 | 0.03074 | 0.74355 | 0.87962 |
| 1D0509-16 | *Gambierdiscus* sp. type 4 | 5 | 18 | 12 | 0.02357 | 0.86957 | 0.95285 |
| 1D0510-22 | *Gambierdiscus* sp. type 4 | 3 | 21 | 17 | 0.05074 | 0.81995 | 0.9203 |
| DS0511-03 | *Gambierdiscus* sp. type 5 | 4 | 21 | 16 | 0.02192 | 0.82526 | 0.92747 |

*RSS*: Residual sum of squares

DF: degrees of freedom
