# Supplementary material for: Influence of Environmental Variables on Gambierdiscus spp. (Dinophyceae) Growth and Distribution
Source: PLoS One. 2016 Apr 13;11(4):e0153197. doi: 10.1371/journal.pone.0153197 (PMC4830584; doi:10.1371/journal.pone.0153197)
Supplement: S2 Table — (DOC) [file pone.0153197.s002.doc]

S2. Growth rate statistics calculated with polynomial fit of *Gambierdiscus* growth rates in response to temperature (16-38ºC).

| **Strains** | **Species** | **polynomial order** | **Number of Points** | **DF** | ***RSS*** | **Adj. R-Square** | **R Value** |
| --- | --- | --- | --- | --- | --- | --- | --- |
| BP Mar10_6 | *G. belizeanus* | 4 | 36 | 31 | 0.04597 | 0.89729 | 0.95343 |
| BP Mar10_7 | *G. belizeanus* | 5 | 39 | 33 | 0.02746 | 0.93663 | 0.9721 |
| BP Mar10_22 | *G. belizeanus* | 4 | 33 | 28 | 0.03505 | 0.93912 | 0.973 |
| FC Dec10_13 | *G. belizeanus* | 2 | 33 | 30 | 0.17814 | 0.72606 | 0.86208 |
| BP Aug08 | *G. caribaeus* | 4 | 42 | 37 | 0.04446 | 0.90158 | 0.95456 |
| FC Nov09_4 | *G. caribaeus* | 5 | 42 | 36 | 0.10708 | 0.7341 | 0.87551 |
| SH Nov09_3 | *G. caribaeus* | 5 | 45 | 39 | 0.12363 | 0.86001 | 0.9359 |
| SH Mar10_12 | *G. carolinianus* | 4 | 27 | 22 | 0.00932 | 0.95622 | 0.9813 |
| BB Apr10_3 | *G. carolinianus* | 5 | 33 | 27 | 0.03654 | 0.80173 | 0.91253 |
| BP May10_1 | *G. carolinianus* | 4 | 33 | 28 | 0.06444 | 0.67926 | 0.84814 |
| KML1 | *G. carpenteri* | 4 | 39 | 34 | 0.02706 | 0.84229 | 0.92676 |
| 3S0509-27 | *G. pacificus* | 4 | 36 | 31 | 0.09563 | 0.83786 | 0.92542 |
| 3S0510-19 | *G. pacificus* | 4 | 33 | 28 | 0.05374 | 0.83584 | 0.9254 |
| FC May10_9 | *G. silvae* | 3 | 27 | 23 | 0.04755 | 0.73583 | 0.87539 |
| 1D0509-16 | *Gambierdiscus* sp. type 4 | 2 | 27 | 24 | 0.03967 | 0.76681 | 0.88586 |
| 1D0510-22 | *Gambierdiscus* sp. type 4 | 4 | 33 | 28 | 0.03861 | 0.92133 | 0.96497 |
| DS0511-03 | *Gambierdiscus* sp. type 5 | 5 | 30 | 24 | 0.01223 | 0.93016 | 0.97067 |

*RSS*: Residual sum of squares

DF: degrees of freedom
